# Century-long butterfly range expansions in northern Europe depend on climate, land use and species traits

**DOI:** 10.1038/s42003-023-04967-z

**Published:** 2023-06-03

**Authors:** Johanna Sunde, Markus Franzén, Per-Eric Betzholtz, Yannick Francioli, Lars B. Pettersson, Juha Pöyry, Nils Ryrholm, Anders Forsman

**Affiliations:** 1 Department of Biology and Environmental Science, Linnaeus University, SE-39182 Kalmar, Sweden; 2 Biodiversity Unit, Department of Biology, Lund University, SE-22362 Lund, Sweden; 3 Finnish Environment Institute (SYKE), Nature Solutions, Latokartanonkaari 11, FI-00790 Helsinki, Finland; 4 Department of Electronics, Mathematics and Natural Sciences, Faculty of Engineering and Sustainable Development, University of Gävle, SE-80176 Gävle, Sweden

**Keywords:** Biodiversity, Biogeography, Climate-change ecology

## Abstract

Climate change is an important driver of range shifts and community composition changes. Still, little is known about how the responses are influenced by the combination of land use, species interactions and species traits. We integrate climate and distributional data for 131 butterfly species in Sweden and Finland and show that cumulative species richness has increased with increasing temperature over the past 120 years. Average provincial species richness increased by 64% (range 15–229%), from 46 to 70. The rate and direction of range expansions have not matched the temperature changes, in part because colonisations have been modified by other climatic variables, land use and vary according to species characteristics representing ecological generalisation and species interactions. Results emphasise the role of a broad ecological filtering, whereby a mismatch between environmental conditions and species preferences limit the ability to disperse and establish populations in emerging climates and novel areas, with potentially widespread implications for ecosystem functioning.

## Abstract (web summary)

Century-long data on climate and butterfly distribution show that species richness has increased with increasing temperature, and that the rate and direction of range expansions are influenced by land use, species interactions and species traits.

## Introduction

Biodiversity is challenged worldwide by environmental modifications brought about by ongoing climate change, a growing human population, invasive species, altered land use, and exploitation, and the impact is expected to continue to increase for the foreseeable future^1–3^. In response to altered conditions, species and populations can move to more suitable areas, adapt, or in the worst case, go extinct^4–12^. Range shifts have been documented in a variety of taxonomic groups^1,6,13–15^. Such rearrangements may change species richness and composition of communities, functioning and resilience of ecosystems, and the services that they provide^7,8,16–18^. While it has been shown that range shift rates vary among taxonomic groups and differ according to study duration, time period and latitude, as well as between species within studies (reviewed in^14^), current knowledge is largely based on analyses of relatively recent changes in restricted areas^19–24^. The mechanistic underpinnings of the observed variability remain largely unknown.

Butterflies are highly mobile, ectothermic animals with short generation times and high fecundity, and are therefore well suited for exploring how range shifts change in response to altered conditions^6,25^. Although it is generally accepted that butterflies are relatively successful in tracking changing climates^26^, there are reports of butterfly species that have not shifted their ranges^27,28^, have lagged behind temperature shifts^21^, and even expanded their ranges in a direction opposite to that of the temperature movement, resulting in mismatches between preferred and available temperatures^1,6^. More recent work indicate that changes in species distributions and shifts in community composition are partly influenced by a complex interplay of climate change^29^, modified human land use practices^12,20,30^, and species-specific characteristics that can affect the capacity to adapt, disperse and establish in novel environments^10,14,28,31,32^. Range expansions likely also depend on species richness and trait distribution in the receiving community that, alongside abiotic factors, might impact community assembly through ecological filtering via species interactions^33,34^. A complete understanding of how all these drivers and constraints contribute to variation in species range shifts requires that they are evaluated using data that covers large spatial scales and long time periods.

**Fig. 1:**
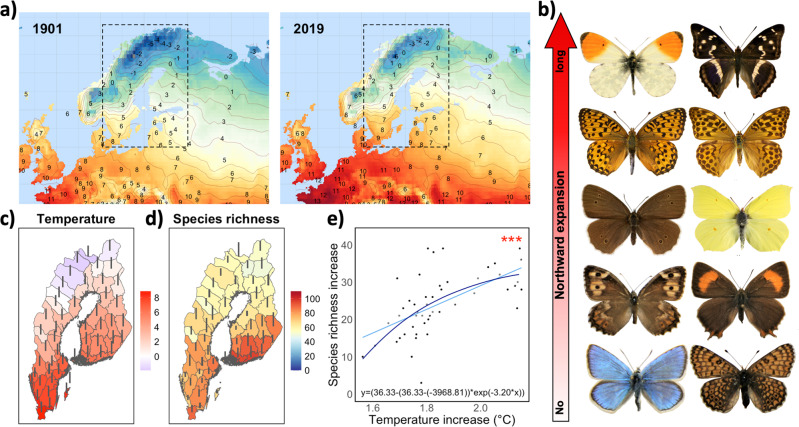
Changes in species richness and temperature from 1901 to 2019 in the study area and examples of butterfly study species. The two top left panels (**a**) show mean annual temperatures in (parts of) Europe in 1901 and in 2019. The right panel (**b**) shows examples of study species sorted from bottom to top according to their northward range expansion (from no to long). *Polyommatus dorylas*, *Melitaea cinxia*, *Hipparchia semele*, *Thecla betulae*, *Aphantopus hyperantus*, *Gonepteryx rhamni*, *Speyeria aglaja, Argynnis paphia*, *Anthocharis cardamines* and *Apatura iris* (Photos by Vladimir Kononenko, reprinted with permission from copyright holder Leif Aarvik). The bottom panel, (**c**) shows current temperature (°C, indicated by colour) and temperature change (1901–2019) indicated by the length of the bar) for each of the 51 provinces in Sweden and Finland, (**d**) shows current species richness (colour) and change (1901–2019) in species richness (length of the bar), and (**e**) shows the linear (light blue line) and asymptotic (dark blue line) increase in species richness with temperature increase from 1901 to 2019. Each dot represents one province (*n* = 48).

**Fig. 2:**
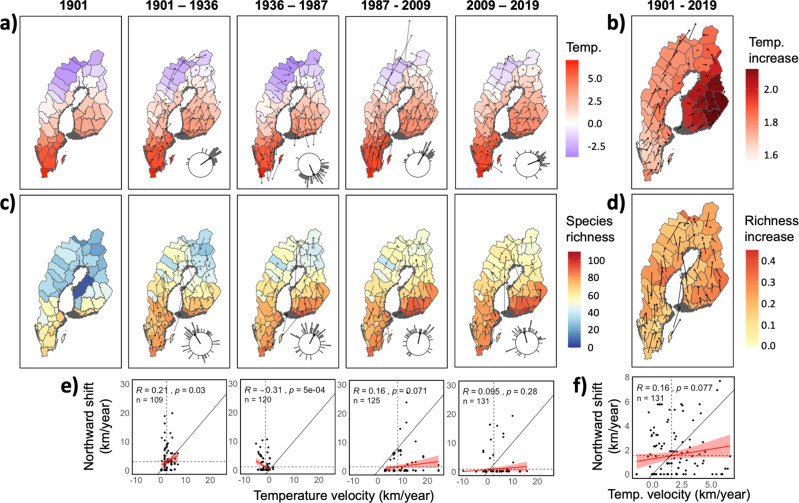
Temperature, average temperature movements, species richness, average species movements, and associations between northward range shifts and northward temperature shifts in four periods during the past 120 years (from 1901 to 2019). Colour codes in the top maps indicate spatial variation in (**a**) temperature (°C) for each timepoint, and in (**b**) temperature increase (°C) over the entire study time range (1901–2019). Colour codes in the lower maps indicate spatial variation in (**c**) species richness for each timepoint and (**d**) species richness increase (species per year) over the entire study time range (1901–2019). Arrows in maps represent mean province based movement per period and over the entire study duration for mean annual temperature (a and b), and species richness (**c** and **d**). Circles in (**a**) indicate the distribution of mean direction of province-based temperature movements (bars, where 0° is north with rotation clockwise, and length of bar indicate the number of observations), and needles from centres indicate the average mean across provinces. Circles in (**c**) indicate the distribution of mean direction of province-based species movements (bars, where 0° is north with rotation clockwise, and length of bar indicate the number of observations), and needles from centres indicate the average mean across provinces. The bottom row shows associations between northward species range shifts (km/year) and northward temperature shifts (km/year) for (**e**) each period separately and (**f**) for the entire study duration. In (**e**) and (**f**), each species contributed one measure of northward range shift velocity per period (calculated as the northward distance (km) between the northernmost province of a species distribution from one time point to the next divided by the duration of the period (in years), for details see Methods), and the corresponding temperature shifts were extracted based on the starting coordinates (longitude and latitude) of each range shift (for details see Methods). *n*-values denote sample sizes, which increased for each period as new species were observed (colonised the study area). Diagonal lines indicate the isometric relationship expected if the rate of species northward range shifts coincide with the rate of temperature shifts. Red lines with shaded areas represent regression lines with 95% confidence intervals. Observations for species with zero northward range expansion have been jittered along the y-axis. *R*-values denote Pearson correlation coefficients.

**Fig. 3:**
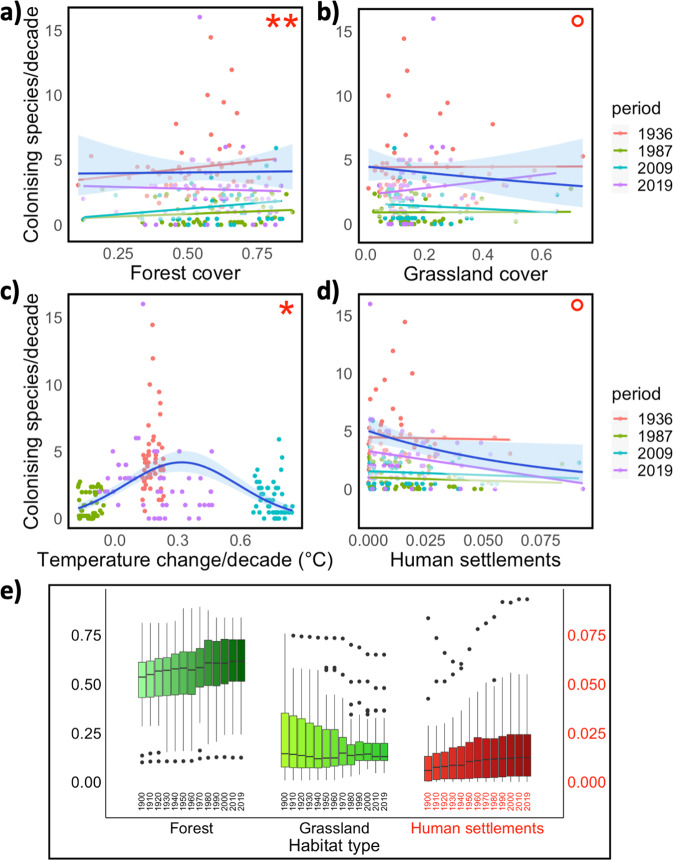
Associations of variation in provincial colonisation rate of butterflies with land use and temperature change. Scatterplots show provincial colonisation rate (number of colonising species per decade) as a function of (**a**) forest cover, (**b**) grassland cover, (**c**) temperature change, and (**d**) human settlements. Each dot represents data for one province per period with different colours of the dots for each of the four time periods. Dark blue lines and blue shading illustrate the predicted mean lines with 95% CI obtained from the GLMM (*n* = 201). The thin trend lines of different colours represent the associations based on linear regressions as estimated separately for each of the four time periods. Red asterisks indicate significant interactions with time period, °0.05 < P < 0.1, *P < 0.05, and **P < 0.01, see Table 1a and Table S1 for details. Bottom panel (**e**) shows how the proportion of each habitat type changed over time (one box every 10th year between 1900 and 2010, and one box for 2019). Each province contributes one value per time point. Boxes indicate interquartile range (IQR), vertical line denote median, upper, and lower whisker third quartile +1.5 × IQR and 1st quartile −1.5 × IQR range, and dots represent outliers. Note that the scale on the vertical axis is different for human settlements. Data for (**e**) were obtained from the HILDA GIS product^102–104^.

**Fig. 4:**
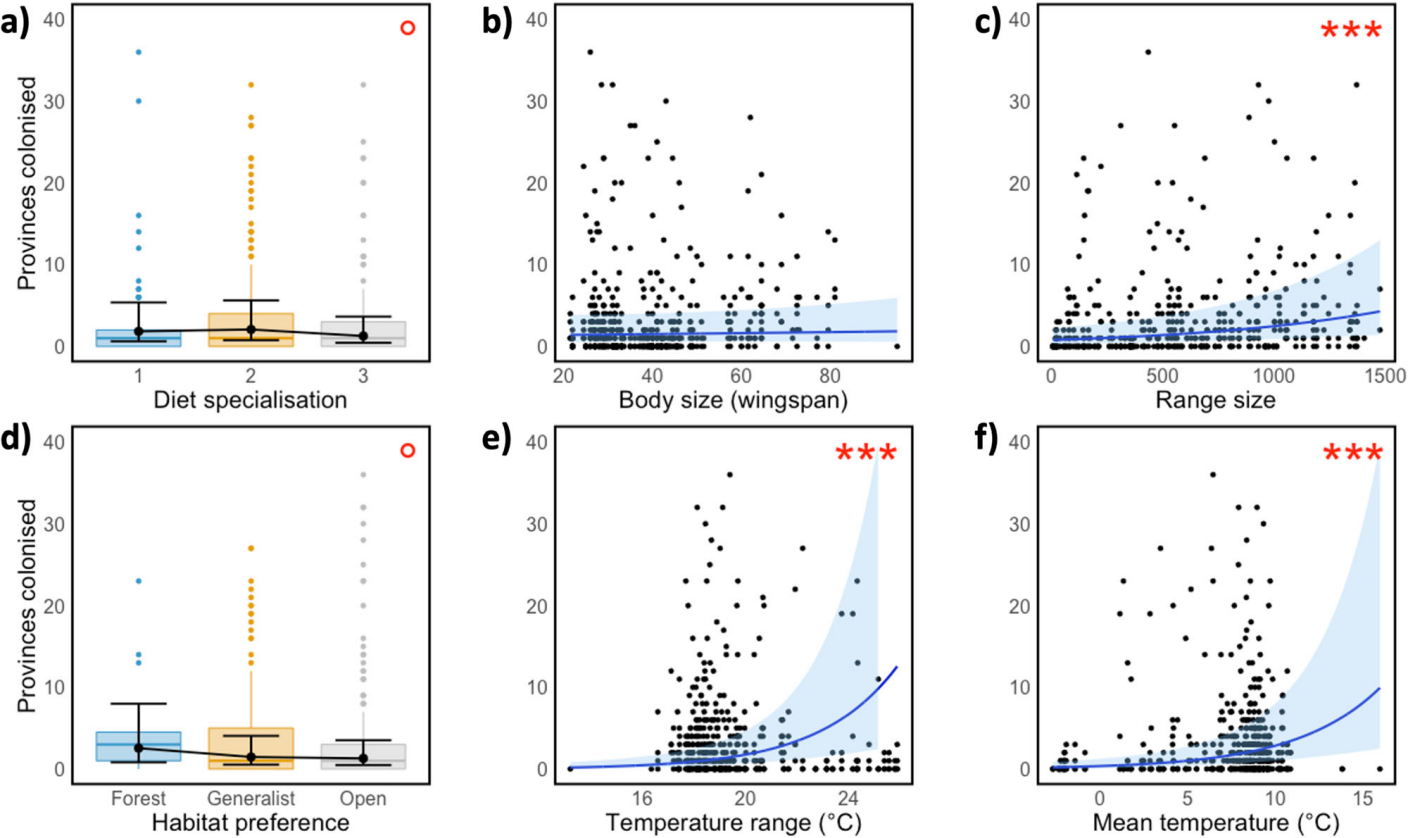
Associations of establishment success of butterflies with species traits. Figures are based on the GLMM and raw data illustrating the number of new provinces the species colonised as a function of (**a**) diet specialisation (where 1 = monophagous, 2 = oligophagous, and 3 = polyphagous), (**b**) body size (wingspan), (**c**) total range size, (**d**) habitat preference, and thermal niche quantified as (**e**) mean species temperature index, and (**f**) range of the species temperature index. Each dot represents one species and one period. The coloured box plots (in **a** and **d**) are based on raw data and boxes indicate the first and third quartiles, horizontal lines indicate the median, vertical lines indicate 1.5 × IQR, and coloured dots indicate outliers. Overlayed in black (**a** and **d**) are least-squares means with associated error bars from the GLMM reported in Table 1b. In panels **b**, **c**, **e**, and **f**, the black dots represent raw data (each dot representing one species and one time period), and the blue lines and shaded areas show the predicted mean lines with 95% CI obtained from the GLMM (*n* = 467) reported in Table 1b. °0.05 < *P* < 0.1, ***P < 0.001, see Table 1b and Table S2 for details.

**Fig. 5:**
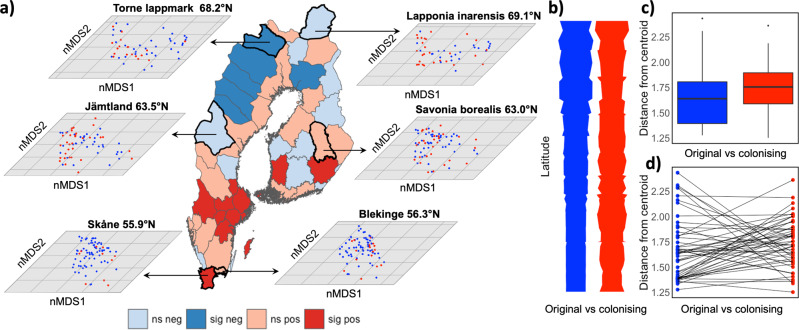
Comparison of within-group variability of trait values between the original community and the colonising species. Within-group variability was estimated (using the PERMDISP implementation in the ‘vegan’ package in R) as the mean dispersion from the centroid based on data of the continuous numeric variables body size (wingspan), species mean temperature, species temperature range, and distribution range size. Left panel (**a**) shows the PERMDISP results from within-province comparisons. Colours in the map indicate whether trait variability was higher in original (blue) (northern) or colonising species (red) (south central), and shades indicate whether the pairwise test was significant (darker shade) or not (lighter shade). Colours in the nMDS plots for a subset of the provinces at three latitudes are blue: original species, red: colonising species. In (**b**) the blue and red vertical bars show the association of within-group trait variability with latitude (wider bars indicate higher trait variability) in the original community (blue) and in the group of newly established species (red). Scatterplots of these relationships are available in Figure S5. The upper right panel (**c**) shows the overall result from the paired comparisons of within-group trait variability (distance from centroid) between original and colonising species in each province. Boxes indicate interquartile range (IQR), vertical line denote median, upper and lower whisker third quartile +1.5 × IQR and 1st quartile −1.5 × IQR range, and dots represent outliers. The lower right panel (**d**) shows the heterogeneity of the outcomes of the province-based pairwise comparisons.

The overall aims of this study are to investigate more than century-long range expansions of butterflies in Sweden and Finland in northern Europe and to holistically examine whether and how these changes depend on climate, land use, species traits of colonisers, and ecological filtering imposed by species interactions. We hypothesise that spatial variation and temporal shifts in temperature will affect species richness and range expansions, and that there will be mismatches in the responses that are driven in part by land use, in part by variation in species traits related to dispersal capacity, thermal niche, and ecological generalisation, and in part by ecological filtering imposed by trait distributions in the receiving community. To evaluate the different components of this overarching hypothesis, we first use distributional data for 131 species of butterflies in 51 provinces in Sweden and Finland (spanning 14.8 latitudinal degrees and 20.6 longitudinal degrees) to reconstruct how the spatial variation in species richness (the cumulative number of species recorded in each province up to the specific time point) has changed from 1901 to 2019 and compare these shifts with temperature changes in the study area during the same period (Fig. 1a–e). Next, we explore whether spatiotemporal variation in colonisation rates was associated with temperature shifts (Fig. 2a–f), land use (forest cover, grassland cover and human settlements), and land use change (Fig. 3a–e). We also evaluate whether and how differences in range expansion between species varied according to species characteristics (i.e., body size, diet specialisation, habitat preference, mean and range of species thermal niche, and European range size; Fig. 4a–f). Finally, we investigate whether establishment of species in new provinces was associated with species richness and community-wide trait distribution in the receiving communities, to indirectly assess the role of species interactions for community assembly (Fig. 5a–d). The specific hypotheses, where appropriate, are presented in the results and discussion sections below.

## Results and Discussion

### Current patterns and past changes

We identify significant temporal shifts in cumulative butterfly species richness since the beginning of the 1900s. The total number of species in Sweden and Finland increased from 108 to 131 between 1901 and 2019, corresponding to a 21% gain. The increase in cumulative species richness (i.e., the number of new species from 1901 to 2019) varied among provinces (3–39 new species per province), with an average increase across all provinces of 24 species (from mean provincial richness of 46 species in 1901 to 70 in 2019), corresponding to an average richness gain of 64% over the period (range 15–229% provincial gain). To our knowledge no butterfly species has gone nationally extinct from these countries during the study period, although there are indications that it may have happened after the end of the study^35,36^. However, several species have declined in abundance and experienced local extinctions (extirpations) owing to habitat degradation and loss^37,38^. Analyses of contemporary butterfly communities in Sweden and Finland using province data for 2019 show that species richness is negatively associated with latitude (also when accounting for spatial autocorrelation, slope estimate ± s.e., −2.30 ± 0.72, *z* = −3.18, *n* = 51, *P* = 0.0015) and positively associated with current temperature (2.62 ± 0.81, *z* = 3.23, *n* = 51, *P* = 0.0012) (Fig. 1c, d). This conforms well with the large-scale latitudinal biodiversity gradients reported in various taxa^39^, and likely derives from covariances among latitude and spatially patterned environmental variables, including components of climate^40–42^.

Our analyses show that the current richness gradient reflects changes in species range expansions that are only partly attributable to climate change. In our study area, climate has changed over the last century with an overall northward (mean annual) temperature movement of about 3 km per year (300 km in the last century), and with the rate of increase varying both among provinces and time periods (Fig. 1a, c, and Fig. 2a, b). This is more than twice the average climatic isotherm shift in Europe and North America (120 and 105 km respectively in the last century)^43,44^, and the faster increase in northern regions is consistent with what has been previously predicted and reported^26,45,46^. In absolute terms, the annual mean temperature has increased by >2 °C in some provinces in our study area (Fig. 2b), which is on par with the projection of future climate change for the next 100 years (IPCC). Next, we explore whether and how these temperature changes have impacted species range expansions and butterfly biodiversity.

### Associations of biodiversity shifts and range expansions with temperature change

Since 1901, species richness has increased with increasing temperature increase (linear regression, estimate ± s.e.: 31.67 ± 6.09, *z* = 5.20, *n* = 48 (three provinces excluded due to lack of data, see Methods for details), *P* < 0.0001), but with the rate of species richness increase levelling off at higher temperature increases (asymptotic regression, estimated plateau ± s.e.: 36.33 ± 6.64, *t* = 5.47, *n* = 48 (three provinces excluded due to lack of data, see Methods for details), *P* < 0.0001) (Fig. 1e), suggesting that the positive effect on biodiversity gradually diminishes as the temperature increases very fast. Within this general pattern of increased species richness over time, there was substantial spatial variation within the study area. For example, species richness increase was positively correlated with latitude (i.e., larger richness increases were found on higher latitudes; with spatial autocorrelation, 1.03 ± 0.42, *z* = 2.45, *n* = 48 (three provinces excluded due to lack of data, see Methods for details), *P* = 0.014), and decreased with increasing average temperature (i.e., lower richness increases at higher average temperatures; with spatial autocorrelation, slope estimate ± s.e., −1.02 ± 0.52, *z* = −1.96, *n* = 48 (three provinces excluded due to lack of data, see Methods for details), *P* = 0.0499).

To evaluate the hypothesis that species richness shifts and range expansions have been driven by spatiotemporal temperature shifts we compared their rates and directions. Northward range expansion rates of butterfly species varied by a factor of three among periods, ranging from ca 3 km/year (on average across species) between 1901 and 1936 to ca 1 (0.9) km/year between 2009 and 2019 (Fig. 2c–e). The choice of time points was based on data availability, as defined in province records (for details see Methods). For each of the five time points, distributional data of Finnish and Swedish butterflies had been compiled and published in national province level catalogues. The results showed that the expansion rates of butterflies have been slower than the shifts in temperature, which varied in both direction and velocity from ca 8 km/year northward between 1987 and 2009 to ca −1.5 km/year (i.e., southward) between 1936 and 1987 (Fig. 2a). Despite variability of temperature shifts, species have advanced their ranges northwards on average during all periods (Fig. 2c). However, this pattern was not universal, and both average rate of observed range shifts and estimated association across species between northward range expansion rates and northward temperature shifts varied among periods (effect of the interaction between period and temperature velocity, Wald ***χ***^2^ = 10.58, *df* = 3, *P* = 0.014, Fig. 2e). During the first period (1901-1936) the rate of species northward shifts corresponds well with the rates of temperature shifts (*r* = 0.21, *P* = 0.03, *n* = 109). Between 1936 and 1987 the rate of range shifts was negatively associated with the rate of temperature shifts (the northward expansions continued despite that temperatures retracted) (*r* = −0.31, *p* < 0.001, *n* = 120), an unexpected outcome possibly reflecting a time lag in the responses to climate change, as previously reported for butterflies^6,21,47^ and other organisms^13,21,48^. Also, summer temperatures were still relatively high in the period 1936-1960 which allowed expansions of many butterflies to continue well into 1950s^49^.

In the last two periods (1987–2009, and 2009–2019), range shifts did not keep up with temperature shifts (1987–2009: *r* = 0.16, *P* = 0.07, *n* = 125; 2009-2019: *r* = 0.095, *P* = 0.28, *n* = 131) (Fig. 2e). When the entire study time range (1901–2019) is considered, the average rate of northward range expansions was not associated with the average rate of the northward temperature shift (*r* = 0.16, *P* = 0.077, *n* = 131) (Fig. 2f). We conclude that rates of butterfly range expansions are only weakly (at best) associated with temperature increments, perhaps in part because climate change besides temperature changes brings with it modifications of seasonality, precipitation and aridity that may also affect species range expansions^7,8,46^. This inability to track the pace and direction of temperature change probably reflects that species range shifts are underpinned by multiple interactive processes^21,48,50^. The weak association might also reflect the inability to capture short-term variation due to the relatively long duration of the time periods. An additional explanation for the weak association is that temperature has not invariably moved northwards; the direction of temperature movements has varied both between periods and provinces (Fig. 2a). Next, we therefore evaluate whether the direction of range expansions co-vary with the direction of temperature change.

The average direction of butterfly range shifts per province from 1901 to 2019 was towards the north (α = −3.2° (356.8°), 0° is north with rotation clockwise, Fig. 2c, d). The mean direction of the temperature shift was towards north-east (α = 36.6°, Fig. 2a,b) and differed from the mean direction of species’ range expansions per province (Watson–Williams test, entire study time range: *F*_1,98_ = 11.49, *P* = 0.001). This mismatch was also apparent when shifts were analysed at higher temporal resolution; separate analyses of each period showed that the direction of temperature and species shifts was significantly different for all of the four periods (1901–1936: *F*_1,96_ = 30.38, *P* < 0.0001; 1936-1987: *F*_1,72_ = 108.01, *P* < 0.0001; 1987–2009: *F*_1,70_ = 5.14, *P* = 0.027; 2009-2019: *F*_1,74_ = 22.08, *P* < 0.0001; samples sizes vary because all provinces did not have species that shifted their ranges in all time periods). Information on directions is provided in Fig. 2a–d. The northward direction of species range shifts over the entire study time range may in part reflect the latitudinally extended shape of our study area. However, such a potential artefact should also affect the estimates of temperature movements, and yet the two are largely independent. In conclusion, we find that the spatiotemporal variation in the average direction of butterfly range expansions per province has not coincided overall with the direction of temperature movements.

The above results raise several important questions. Why did range shifts of butterflies not keep up with the rate or match the direction of the temperature shifts? What were the underpinnings that contributed to the variability in magnitude and direction of range expansions between periods? Our data also show that some species have moved much faster (e.g., *Apatura iris*), some have failed to keep up with temperature change (e.g., *Melitaea cinxia*), and some species have matched the temperature shifts (e.g., *Nymphalis polychloros*). This spurs the question: what were the reasons for the heterogeneity of responses among species? To answer these questions, we next evaluate the hypothesis that range expansions are modified by variation and change in land use and vary according to differences in species traits.

### Evaluating the contribution of land cover and modified land use

Many aspects of land use in Sweden and Finland have changed during the past 120 years (Wald ***χ***^2^ = 15.65, *df* = 4, *P* < 0.004) (Fig. 3f, Fig. S1). Specifically, separate analyses of each land use category showed that since 1900, the proportional area covered by forest has consistently increased, particularly in central and northern provinces (Wald ***χ***^2^ = 206.37, *df* = 4, *P* < 0.0001). Grassland has instead decreased in northern provinces and increased slightly in central and southern provinces (Wald ***χ***^2^ = 27.65, *df* = 4, *P* < 0.0001). In addition to the proportional cover, the quality of forest and grassland habitats have also changed. Modified silviculture practices with shorter harvest intervals have resulted in more frequent and longer periods of temporary open habitats, which are colonised and utilised by butterflies as well as birds^51–53^. These suitable areas are nowadays available during a large proportion of the forest cycle, and rather than being uninhabitable to most butterflies, forests appear to have a growing potential as a complimentary habitat^51^. In many cases, grasslands have transformed into forests through the process of succession, particularly when farms are abandoned, and especially in the northern regions. In the southern areas, most grasslands are heavily fertilised fields used for the production of silage, and this type of habitat is less suitable for butterflies compared with traditional hay meadows with late harvest^38^. Human settlements have also increased, particularly in southern provinces (Wald ***χ***^2^ = 54.19, *df* = 4, *P* < 0.0001) (Fig. S1).

Our analyses support the hypothesis that this variation in land cover and changes in land use over time reported above accounts for some of the spatiotemporal variation in butterfly range expansions and community species richness. Results show that the provincial colonisation rate (number of species per decade colonising the province) differed between periods, and further that the associations of colonisation rate with land use and land use change (forest cover, grassland cover, and human settlements) varied among the periods (Table 1a, Table S1, Fig. 3a–d). Specifically, the associations of colonisation with forest cover varied through time (effect of interaction with time period, *P* = 0.010) and changed from positive to neutral during the last period (2009-2019) (Fig. 3a). In contrast, although not below the traditional alpha-level (*P* = 0.100), the association of colonisation rate with grassland cover changed from neutral to positive during the last period (Fig. 3b). These findings further emphasise that the consequences for biodiversity of climate change and modifications of land use are context specific. The provincial colonisation rate tended to decline with increasing urbanisation (i.e., increasing human settlements), particularly during the last period (interaction between period and human settlements: *P* = 0.081; Table 1a, Table S1, Fig. 3d), which is in accordance with existing evidence that urbanisation reduces and homogenises biodiversity^32,54–56^, although some studies suggest that urban areas can have higher diversity than nearby natural areas^57–59^. Land use, including agriculture, is less intense in northern than in southern regions where eutrophication and pollution, along with urbanisation have their strongest impacts^60^. It is therefore possible that the intensity of land use in the southern regions may reduce colonisation rates^60^.

**Table 1:** Associations of provincial colonisation rate and species establishment success in butterflies with environmental factors and species traits.

(a)	Predictor	Wald *χ*^2^	*df*	*P*-value	(b)	Predictor	Wald *χ*^2^	*df*	*P*-value
	(Intercept)	7.01	1	0.008**		(Intercept)	2.71	1	0.099
	Forest	0.01	1	0.907		Diet specialisation	5.35	2	0.069
	Grassland	0.01	1	0.918		Body size (wingspan)	0.40	1	0.530
	Temp change (linear and squared)	10.54	2	0.005**		Range size	15.08	1	<0.001***
	Human settlements	0.73	1	0.392		Temperature range	13.09	1	<0.001***
	Period	10.10	3	0.018*		Mean temperature	13.95	1	<0.001***
	Forest x Period	11.36	3	0.010**		Habitat preference	4.39	2	0.111
	Grassland x Period	6.26	3	0.100					
	Temp change (linear and squared) x Period	16.37	6	0.012*					
	Human settlements x Period	6.74	3	0.081					

(a) Results from a generalised linear mixed model for associations of provincial colonisation rate (number of colonising species per province and decade) with period, land use and temperature change. Province (var = 3.23 × 10^−70^, sd = 1.80 × 10^−35^) was included as a random variable. (b) Results from a generalised linear mixed model for associations of establishment success (number of new provinces colonised) with species traits. Relatedness (species nested in family) (var = 0.47, sd = 0.68) and period (var = 0.98, sd = 0.99) were included as random variables. The outputs from the ‘summary’ function are available in Tables S1 and S2.

The associations with land use reported above manifested despite the fact that the effect of temperature change on colonisation was accounted for in the statistical model (Table 1a). It is noticeable that the association with temperature change was curvilinear (Table S1), with colonisation rate peaking at around 0.30 °C increase per decade (Fig. 3c). This indicates that, at least in our study area and during this study period, an intermediate rate of temperature increase has promoted the establishment of new species, whereas more drastic temperature increments have had a less favourable impact on species range expansions, likely due to time-lags and climatic debt^6,13,61^. This adheres to the asymptotic increase in species richness with temperature change observed during the entire study time range (Fig. 1e). In conclusion, these results suggest that external environmental conditions, such as climate change (Fig. 1a) and modified land use (i.e., increased forest cover, decreased grassland, and urbanisation Fig. 3e) impact the ability of butterflies to colonise new areas.

### Evaluating the contribution of species traits

Besides the role of ecological filtering imposed by external environmental conditions such as land use and climate, the ability of butterflies to colonise new areas in pursuit of favourable conditions also varies according to species attributes. Northward range expansion rates and establishment success varied considerably among species (Fig. 1b, Fig. 4a–f, Table 1b). This may to some extent reflect differences in evolutionary potential and adaptability^10,11,28^. Although the role of adaptations cannot be evaluated with the data available in this study, earlier work report on several butterfly species that have been observed to alter their habitat preferences and larvae diet when expanding their ranges (e.g.^62–64^,). The likelihood of colonisation and establishment success may also vary according to differences in species traits related to dispersal capacity, ecological generalisation and the ability to cope with variable and novel conditions^14,28,65,66^, such as range size, thermal niche, larval diet breadth, habitat use and body size. Our analyses support this hypothesis. Specifically, the establishment success of species (the number of new provinces colonised each period) was associated with four of the six species traits investigated (Table 1b, Table S2, Fig. 4a–f). Species establishment success increased with European range size (*P* < 0.001), and with both the mean (*P* < 0.001) and the range (*P* < 0.001) of species temperature index (Table 1b, Fig. 4c, e–f). The establishment success increased about equally fast with the range (width quantified as difference between the annual warmest month and the annual coldest month; slope estimate = 0.73, sd = 0.20) as with the mean (slope estimate = 0.74, sd = 0.20) of the species thermal niche (Table S2). Indeed, by limiting which environments that can be occupied, the species’ thermal niche seems to be driving range expansions and influencing the structure of ecological communities across biomes^19,21,41,67–69^. Together, these findings support the hypothesis that generalist species with broad niches are better able to cope with new and changing conditions^70–72^.

In addition, the association between establishment success and diet was marginally significant (*P* = 0.069), suggesting that generalist species utilising several larval host plants expanded less than species specialising on one or a few host plants (Table 1b, Fig. 4a, Table S2). This outcome might seem counterintuitive, but likely reflects that diet generalists were more widespread already at the beginning of the study period and therefore could not realise their higher capacity for colonisation, whereas species with narrow diets and small ranges were able to expand their distributions. In support of this interpretation, monophagous, oligophagous, and polyphagous butterfly species occupied on average 10, 18 and 24 (of the 51 provinces in total) in 1901, respectively. It is also worth noting here that the establishment success of species was not correlated with their initial occupancy (number of provinces occupied; see Fig. S2), indicating that the associations of establishment success with range size, thermal niche and diet reported here were not confounded.

Although the overall association between establishment success and habitat preference was not below the traditional alfa-level of 0.05 (*P* = 0.11, Table 1b, Fig. 4d), pairwise comparisons showed that forest specialists expanded their ranges more than open habitat species (*P* = 0.036, Table S2), with the magnitude of the difference corresponding to about three (2.6) versus one (1.3) (based on least-squares means) new provinces colonised per period, or about eleven (10.5) versus eight (8.0) (based on medians) new provinces colonised during the entire study time range (1901–2019). The relative success of forest specialists, compared with open habitat species, conforms well with the increased coverage of forest throughout the study period and the decreased coverage of grassland in the northern part of the study area (Fig. 3e, Fig. S1). Species establishment success was not associated with body size (wingspan) (*P* = 0.53, Table 1b), contrary to some^28^ and in agreement with other previous studies^31,73^.

### Evaluating the contributions of species richness and community-wide trait distributions

As we have seen, range expansions have been influenced by climate warming, land cover, modified land use and species traits. Our results reported below further suggest that the potential for species to establish new populations and expand their ranges was influenced also by ecological filtering during the community assembly process mediated in part by the species in the recipient community^74,75^. This was evidenced by that on an overall level, the colonisation rate decreased with original species richness in each province and period (Wald ***χ***2 = 9.05, *df* = 1, *P* = 0.003; Fig. S3), possibly reflecting the fact that it is less likely for a coloniser to count as a new, additional species in provinces with a high original species richness, or that community assembly is influenced by interspecific competition^22,76^. The total gamma diversity in our study area is 131 species, and even the most species rich provinces were far from being filled with all species in 2019 (maximum 96 species with an average richness of 70 species per province)^22,76^. Although we have no information on species abundances in the early 20th century, we assume that higher species richness coincides with more individuals, such that interspecific competition is likely more intense in species rich communities in southern parts of our study area than in the species poor north^77^. However, direct interspecific competition is considered rare among herbivorous insects^78^. That the southern and most species-rich provinces have gained relatively few new species could also reflect that both Finland and Sweden are restricted from continental areas in the south by sea, which effectively prevents colonisations by weaker dispersers. During the study period, the butterfly fauna in Finland and Sweden have increased by 23 new species, primarily in the eastern and southern provinces.

Average multidimensional trait distribution differed between species in original communities and species that colonised new provinces (PERMANOVA, based on the four continuous traits body size (wingspan), species mean temperature and temperature range, and range size: *F*_1, 4776_ = 24.73, *P* < 0.001) (Fig. S4), supporting the conclusion that range expansions were associated with species traits and with species associated to high temperatures colonising more provinces (Fig. 4), see also Faltýnek Fric et al. (2020)^79^. In addition, within-group variability of trait values (dispersions from centroids) was larger on average in colonising species (*F*_1, 4776_ = 21.95, *P* < 0.0001, Fig. 5a–d). That newly colonising species had trait value distributions that overlapped less among species suggests that rare or unique trait value combinations increased the probability of recruiting into the community. The underpinnings cannot be identified with certainty based on the data at hand, but possible mechanisms include better opportunities to occupy empty niches, avoidance of interspecific competition, or that establishment and long-term persistence is improved by ecological generalisation and the ability to utilise novel resources^28,63,71,72,80^. There are also examples of butterfly species that have increased the variety of habitat types that they occupy after having expanded their range^62^. Similarly, Martin et al. (2021)^64^ report that females in edge populations of the *Lycaena dispar* butterfly showed a higher degree of generalism and laid eggs under a wider range of microhabitats, compared with core populations. This might suggest that the relative advantage of generalist versus specialist strategies may change during the course of the range expansion process. Specifically, it can be hypothesised that ecological generalisation promotes initial colonisation and establishment, whereas long-term persistence may require adaptation to local conditions (narrow niches). In support of this last hypothesis, Singer and Parmesan (2021)^63^ report that the diet breadth of the *Euphydryas editha* butterfly first increased after colonisation events as diversification of individual host preferences pulled novel hosts into population diets, but populations that persisted subsequently became more specialised and reverted toward monophagy. To further evaluate these hypotheses, we analysed spatial patterns and trends by comparing trait distribution between colonising and original species in southern (old) and northern (relatively young) communities.

The results of the spatial analyses are in agreement with the hypothesis that broad niches promote range expansions but do not support the notion that persistence requires subsequent specialisation. Groups of colonising species were significantly (*P* < 0.05) more variable compared with the original communities in some south-central provinces, and significantly less variable than original communities in some northern provinces (Fig. 5a). This might reflect that the northern, and relatively young and depauperate communities were originally composed of cold tolerant generalist species capable of coping with strongly seasonal environments^81^. With climate change, the distribution of available resources (e.g., host plants and thermally suitable habitats) has changed, likely allowing for more recent colonisation and establishment in northern provinces also by thermophilic species with different resource demands. Conversely, the more species rich provinces in the south-central part of our study area with less pronounced seasonality may have been more accessible for recruitment by specialised species that rely on rare or unique resources not already utilised by members of the original, and relatively old, community. This explanation, based on the hypothesis that community assembly operates via a filtering process imposed by a combination of abiotic environmental constraints, resource availability and species interactions^33,34,74,75^, is supported by the fact that the effect of latitude on within-group multidimensional trait variability (while controlling for potential confounding effects of province size) differed between the original communities and newly colonising species (interaction between latitude and grouping (original or newly colonised species): Wald ***χ***2 = 14.95, *df* = 1, *P* = 0.0001), and that it increased three times faster with latitude in original communities (slope estimate ± s.e.: 0.069 ± 0.006, *z* = 10.30, *n* = 51, *P* < 0.0001) compared with the variability among colonising species (slope estimate ± s.e.: 0.022 ± 0.01, *z* = 2.18, *n* = 51, *P* = 0.029) (Fig. 5a–b, Fig. S5). These latitudinal trends in community-wide trait distribution are not consistent with the notion that niche breadth should decrease over time. However, this conclusion must be tentative because our approach does not specifically allow for evaluation of evolutionary shifts in intraspecific niche breadth.

The finding that species richness of butterflies decreased with latitude whereas their community-wide trait diversity instead increased with latitude shares some resemblance to the negative latitudinal species richness gradients as opposed to the positive intraspecific (rather than community wide) diversity gradients reported in previous studies^39,82^. The opposing spatial trends of species richness and community-wide trait distribution also conform with the temporal shifts recently reported in moths, with an increase in species richness between 1975 and 2019 being paralleled by a community-wide phenotypic trait homogenisation^83^.

In sum, while climate warming only had limited explanatory power to explain the community-wide trait shifts, instead land cover and land-use change played key roles. These external drivers jointly interact to shift community assembly leading to the emergence of more generalist communities, as witnessed by increases in larger, more generalist, often forest species as well as a higher trait dispersion of colonising species compared to residents.

## Conclusions

Our analyses of spatiotemporal variation in species richness and reconstructed range expansions of butterflies in 51 provinces throughout Sweden and Finland signal an overall dramatic increase in insect biodiversity over the past 120 years in this northern part of Europe. Whilst we note that undocumented provincial extinctions of butterflies might have resulted in overestimations of regional diversity increases^37^, our results were qualitatively robust to the inclusion/exclusion of such potential local extirpations during the study period (see Methods and Supplementary Note 1 in the supplementary materials for details). To our knowledge, no national extinction of any butterfly species has occurred in these countries during the study period^35,36^, and the total species richness shows a 21% gain. This is in sharp contrast to the results and conclusions reported in some previous studies of butterflies and other organisms in different parts of the world, thus further emphasising the context-specificity of responses to global environmental change. As a consequence of the elevated temperatures, new areas, primarily at higher latitudes, that were previously unsuitable, by being too cold, have now become available for colonisation, for example by some thermophilic (and diet specialist) butterfly species. Importantly, that the rate and direction of range expansions have not matched overall the temperature movements reflected that colonisations have been strongly modified by land use and vary according to differences between species in ecological generalisation and thermal niche. Collectively, results emphasise the role of a broad ecological filtering^74,75^, whereby a mismatch between land cover changes, rising temperatures, and species preferences and tolerances appear to limit or delay range expansions. These findings are particularly relevant in the context of the drastic changes in land use and land cover that have occurred in our study region, in particular with regard to the pronounced statistically significant increases in forest cover and human settlements, together with the aforementioned region specific modifications of grassland cover (Fig. 3e). These changes likely interact differently with climate warming, with forests potentially providing more buffering against warming effects compared with for example grasslands. The results further support that the ability of butterflies to colonise new areas in pursuit of favourable conditions increases according to certain species attributes, specifically range size, thermal niche breadth, larval diet breadth, and habitat use, thus ultimately leading to more generalist and species rich communities.

Better understanding of how the interactive effects of shifting temperatures and large-scale changes in land use influence the extinction/colonisation balance and species composition in butterfly communities is now needed to fully appreciate the implications for protection of butterfly biodiversity. This includes systematic evaluations of the role of local adaptations and evolvability of species. The potential for community-wide trait distributions to contribute to variation in establishment success indicated by our results must also be recognised and addressed. Lastly, butterflies interact with several other types of organisms, as competitors, pollinators, prey for dragonflies, spiders and birds, hosts to parasites and parasitoids, and even as predators^29^. There are thus many ways by which changes in the abundance, distribution and structure of butterfly communities may cascade up and down in the ecosystems and affect the services that they provide^84–86^. Ultimately, this can have important implications for forestry, food production, recreation opportunities, human well-being, and connection to nature^16,87–89^.

## Methods

The distribution and distributional changes of butterflies in Sweden and Finland during the last century were analysed in relation to temperature, land use, species richness, and species traits, to determine if butterflies have been able to track climate change and to investigate whether and how the climate, environment and/or species traits influence climate tracking ability. To this end, data on species presence/absence, temperature, and land use for 51 biogeographical provinces, covering the entire Sweden and Finland, was collected for five timepoints (1901, 1936, 1987, 2009, and 2019), and changes during the four periods between the timepoints (1901–1936, 1936–1987, 1987–2009, and 2009–2019) and overall changes (1901–2019) were calculated. The specific time points we used were determined by the data availability for species distributions in the province record catalogues.

### Species data

For each of the five time points, information on species distributions was retrieved from the national province level catalogue of Finnish and Swedish butterflies: 1901^90–92^, 1936^93,94^, 1987^95^, 2009 and 2019. For Sweden, data was curated online 1987, 2009 and 2019^96^. For Finland, provincial data was compiled for 2009 and 2019 by JP based on online sources (https://www.perhoset.fi and https://laji.fi/en) and by updating the data in Kullberg (2002)^97^. As Finland and Sweden have an outstanding tradition to map their fauna and flora, these unique province catalogues reaching back to 1901 contain reliable information with great coverage at the provincial level. To minimise the risk of data bias, taxa that had been split during the study period (*n* = 2, *Plebejus argus* and *P. idas*, *Leptidea sinapis* and *L. juvernica*) were merged to one taxon respectively, thus resulting in a total of 131 butterfly species included. In addition, data on species richness at the first time point and species richness increase during the first period were excluded for three of the Finnish provinces (Om, Oa and St) due to inadequate sampling and province delimitations in the early 1900s. Cumulative species richness for each province at each time point was estimated as the cumulative number of butterfly species observed in the specific province up to the specific time point, species richness increase was calculated as the difference in cumulative species richness from one time point to the next. To make values comparable among the non-linearly divided periods, the rate of species richness increase was also calculated as the cumulative species richness increase divided by the duration of the period (in years or decades). Because the provinces have not been monitored at comparable intensity at regular intervals, at the right time of the season, and during periods with suitable weather conditions it is virtually impossible to identify local extinctions and recolonisation dynamics with high resolution over this large geographic scale and long time period, based on existing province record data. Because of this, and the fact that our results were robust (remained qualitatively similar) to the inclusion/exclusion of species that may possibly have become locally extinct during the study period (see Supplementary Note 1 and Table S3 in the supplementary materials for details), we chose to use cumulative species richness to avoid using potentially unreliable data.

### Range shift velocities and directions

Based on the presence/absence data we estimated the velocity and direction of range shifts. Range shift velocity for each species and time period was calculated as the northward distance (km) between the northernmost province of a species distribution from one time point to the next, divided by the duration of the period (in years).

The directions of the range shifts were estimated based on latitude and longitude. To this end, for each colonisation event we inferred the most likely colonisation route (as the centroid of the closest province in which the species had been observed in the present or previous time points, i.e., assuming the shortest colonisation route). Based on these inferred colonisation routes (vectors), the average (across species) direction of species range shifts was calculated for each province and time period. This procedure thus generated one average species range shift direction (angle) per province and time period.

### Species traits

All species for which range shifts were possible (all but *Aglais urticae*, which occurred in all provinces already at the first time point (1901)) were classified for six species traits: diet breadth, body size (wingspan), range size, mean and range of the species temperature index (STI), and habitat preference. We extracted information on larval diet from the literature^98^ and classified species according to the dietary breadth: specialists (species for which the larva feed mainly on a single plant species), oligophagous (species for which the larva feed on two to five plant species or mainly on a particular genus) or generalists (species for which the larva feed on six or more plant species or on genera in at least two families). Information on adult body size (male wingspan in mm) was collected from the literature^99^. As a measure of species range size (based on distribution data from 1981 to 2000), we extracted information from Schweiger, et al. (2014)^100^ on the number of European 50 × 50 km grid cells occupied. From the same source we also extracted the mean and the range (quantified as the difference between the warmest month and the coldest month) in °C of the species temperature index, which are indicative of the location and continentality of the thermal niche, respectively. Lastly, information on habitat preferences of each species (open, forest, or generalist) was also obtained from the literature^98,99^.

### Temperature data

For temperature calculations we used HadCRUT data from 1900 to 2019 downloaded 2020-08-01 at 1 × 1 degree resolution (gridded yearly global temperature data)^101^. Mean annual temperature for hexagons of 2165 km^2^ was calculated; for the first time point (1901) as the average temperature during the period 1900–1909, and for the last four time points (1936, 1987, 2009 and 2019) as the average temperature during the last 10 years of the specific period. Mean annual temperature for each province was subsequently calculated as the mean of the hexagon temperatures within the specific province. Temperature change for each province and period was calculated as the difference between the mean annual temperature from one time point to the next.

### Temperature shift velocities and directions

Based on the gridded hexagon temperature data, distance and direction of the temperature shifts could be calculated using a slightly modified procedure as that used by Lehikoinen and Virkkala (2015)^48^. To estimate the northward temperature velocity, the mean temperatures for the hexagons were used. For each hexagon and time point (T), we first selected the 100 hexagons closest in temperature in the following time point (T + 1). From the 100 selected hexagons, we then selected the ten that were closest in distance to the starting hexagon. For each of these ten hexagons we computed the vector from the starting hexagon, and then averaged them. This procedure yielded a vector representing the general direction and amplitude of the temperature shift for each hexagon during each period. The northward temperature velocity for each province was defined as the northward component (km) of the average temperature shift of all temperature vectors in the province divided by the duration of the period (in years). Based on the vectors, we also calculated the direction of the temperature shift for each province during each period, as the mean angle of the hexagon temperature vectors for the specific province and period, thus resulting in each province getting one direction/angle of temperature shifts per period.

### Land use data

Data on the proportion of five land cover classes (open land, forest, grassland, human settlements, and cropland) per province was extracted from Historic Land Dynamics Assessment (HILDA) version 2.0 (date of the version is 29-4-15) and the HILDA+ project that is on a spatial resolution of 1 km by 1 km per decade for the period 1900–2019^102–104^.

Climatic variables are often highly correlated, and such collinearities among predictor variables can create model instability and degradation in predictive performance^105^. To avoid problems associated with this, we carried out cluster analysis on the eight ecologically relevant predictor variables (open land, cropland cover, forest cover, grassland cover, human settlements, average temperature, temperature change, and latitude) to assess potential collinearities, and to select the least correlated variables. For this, we used the ‘Varclus’ function in the ‘Hmisc’ package^106^, and a Spearman correlation coefficient threshold of 0.3^107^. This cluster analysis resulted in that out of the eight initially included variables, four (forest cover, grassland cover, human settlements, and temperature change) were retained for the following analyses (Fig. S6).

### Statistics and reproducibility

All statistical analyses were carried out in RStudio2 v.1.3.1093^108^ using R.4.0.3^109^, and data visualisations were created using ggplot^110^ unless otherwise stated. To investigate if butterflies have tracked climate change and whether and how the climate, environment and/or species traits have influenced climate tracking ability, we mainly used linear mixed models (LMMs) and generalised linear mixed models (GLMMs). The different types of models were selected depending on the error distribution of the response variable: LMMs were used for data with normal response distributions (namely species richness, species richness increase, land cover, and within-group-trait variability (multivariate dispersion from centroid)), and GLMMs with a negative binomial fit and a log link function for data with zero-inflated response distributions (namely northwards range shift velocity, provincial colonisation rate, establishment success, and initial occupancy). For these analyses, the ‘glmmTMB’ function in the ‘glmmTMB’ package^111^ was used. Significance of model terms was assessed using Likelihood Ratio tests (type III) implemented in the ‘Anova’ function in the ‘car’ package^112^, and the ‘summary’ function in base R was used to evaluate pairwise differences. Unless otherwise stated, the alpha-level was set to 0.05, and *P*-values < 0.05 were considered significant.

### Contemporary patterns of butterfly species richness

To investigate whether the contemporary patterns of butterfly species richness were associated with latitude and temperature, a separate general LMM was used for each of the two response variables (to avoid problems from collinearity owing to the predictor variables being correlated). For these analyses, province-based data was used; species richness for the last time point (2019) was used as a continuous numeric response variable in both, and either temperature at the last time point (2019) or latitude (centroid of each province) was used as a fixed continuous numeric variable (in the separate models). In addition, to account for province proximity (spatial autocorrelation), which could possibly influence our results, we included a spatial component in the models. This was done by implementing the spatial exponential structure (chosen after comparing the model validation results for all available spatial structures) in the available covariance structures of the ‘glmmTMB’ package. To construct the spatial structure, a Euclidean distance matrix was calculated based on the coordinates of each province (latitude and longitude of centroid). Furthermore, a dummy variable (group = 1) was used in the random structure ‘exp(pos + 0|group)’ to group the coordinates, following Brooks et al. (2017)^111^.

### Associations of biodiversity shifts and range expansions with temperature change

To investigate past changes of butterfly communities, two separate LMMs were used to test whether species richness increase was associated with latitude and temperature. For this, the same type of model as for the species richness tests (described in the previous paragraph) was used, but overall (1901–2019) species richness increase was introduced as the continuous numeric response variable.

To test if overall species richness increase was associated with overall temperature increase a LMM was used. In addition, to investigate whether species richness increased asymptotically with temperature increase, we also used an asymptotic dose-response model (asymptotic regression), ‘drm’ function in the ‘drc’ package^113^. For both of these analyses, province-based data was used; overall (1901–2019) species richness increase (total number of new species) as continuous numeric response variable and overall (1901–2019) temperature increase as a fixed continuous numeric predictor variable. In the test of an asymptotic relationship, the ‘DRC.asymReg’ function was used as the non-linear (asymptotic) function, and statistical significance was assessed with the ‘summary’ function.

To test whether the velocities (rates) of butterfly range shifts was associated with that of the temperature shifts, we used a GLMM in which northward range shift velocity (km/year) was used as a continuous numeric response variable and northward temperature velocity (km/year) as a fixed continuous numeric predictor variable. For this, each species contributed one measure of northward range shift velocity per period, and the corresponding temperature shifts were extracted based on the starting coordinates (longitude and latitude) of each range shift. To evaluate whether the effect of the temperature velocity has differed between the periods, the interaction between temperature velocity and period (introduced as fixed factorial predictor variable) was also included. To calculate Pearson correlation coefficients and to assess statistical significance (for the entire study time range (1901–2019) and for each of the four time periods separately) the ‘stat_cor’ function in the ‘ggpubr’ package^114^ was used.

To test whether the directions of butterfly range shifts were associated with those of the temperature shifts, we used Watson-Williams test (‘watson.williams.test’ function in the ‘circular’ package^115^). For this, each province from which species range expansions had occurred during a period contributed two estimates of directions - one for species range shift and one for temperature shift - and a paired design was used such that the directions were grouped within the provinces. A separate Watson-Williams test was carried out for each of the four time periods, and an additional test was run based on the entire study time range (1901–2019) to investigate the overall effect.

### Evaluating the contribution of land cover and modified land use

To investigate whether land use has changed during the time range, we used LMMs. First, to evaluate the overall effect, the proportional cover of the three habitat types that passed the Varclus analysis (forest cover, grassland cover, and human settlements; for details on variable selection see ‘Land use data’ above) was used as continuous numeric response variable, period as a fixed factorial predictor variable, and habitat type nested in province as a random factorial variable. Next, to further investigate the habitat type-specific effects, an additional LMM was run for each of the habitat types separately, using the same type of model as for testing the overall effect: proportional cover as continuous numeric response variable, period as a fixed factorial predictor variable, and province as a random factorial variable.

To investigate whether and how land use affects the provincial colonisation rate, we used a GLMM. Provincial colonisation rate (number of colonising species per decade) was used as a continuous numeric (integer) response variable, and each province contributed one value per period. The three habitat types (forest cover, grassland cover, and human settlements) and the rate of temperature change (°C per decade; both linear and quadratic relationship) were used as fixed continuous numeric predictor variables, and province was included as a random factorial variable. All predictor variables were normalised (with the ‘scale’ function in base R) to enable comparisons of slope estimates among the predictors. To evaluate if the effect of land use on the provincial colonisation rate differed among periods, we also tested for an interaction effect between land use and period. Predicted means were obtained for each of the predictor variables separately, by dropping the interaction term from the full model and using the ‘effect’ function (xlevels = 1000) in the ‘effects’ package^112,116,117^.

### Evaluating the contribution of species traits

To investigate whether and how establishment success (i.e., species expansion) was influenced by species traits, we used a GLMM. For this, the number of new provinces colonised per species was used as a continuous numeric response variable, and each period contributed one value per species. The six selected species traits were used as either continuous numeric (body size (wingspan), range size, mean and range of the species temperature index) or factorial (diet breadth and habitat preference) fixed predictor variables (for details about the species traits see ‘Species traits’ above). The four continuous numeric predictor variables were normalised (with the ‘scale’ function in base R) to enable comparisons of slope estimates among the predictors. Period and species relatedness (species nested in family) were included as random variables to account for differences among periods and for greater similarity among more closely related species. Predicted means for the four numeric predictor variables (body size (wingspan), range size, mean and range of the species temperature index) were calculated in the same way as for land use data, and least-squares means for each level within the two factorial predictor variables (diet breadth and habitat preference) were estimated using the ‘emmeans’ function in the ‘emmeans’ package^118^.

To also evaluate potential effects owing to differences in initial occupancy, we tested whether the establishment success was associated with the initial number of provinces inhabited. For this, a GLMM based on period-separated data was used. In this, establishment success (number of provinces colonised) was used as continuous numeric response variable, initial occupancy (number of provinces inhabited at the start of period) as a continuous numeric predictor variable, period as a factorial predictor variable, and species as a random variable (to account for differences between species). To test for period-specific effects, the interaction term between initial occupancy and period was also included. Because the test revealed that the effect of initial occupancy did not differ among periods (interaction effect initial occupancy x period: *P* = 0.49), the analysis was rerun excluding the interaction term to assess the main effect of initial occupancy.

### Evaluating the roles of species richness and community-wide trait distributions

To evaluate potential effects of ecological filtering we first used LMMs to investigate whether colonisation of new species in the provinces were associated with the original number of species. This was done twice: once using data for the entire study time range (1901-2019), and once using period-based data. In both analyses, the provincial colonisation rate (new species per decade) was used as a continuous numeric response variable, original number of species (at the start of the entire study time range or period, depending on the analysis) as a fixed continuous numeric predictor variable, and province as a random factorial variable. For the analysis using period-based data, period was also included as a random factorial variable.

To investigate community-wide trait distributions, we compared average multidimensional trait distribution and within-group variability of trait values between the original community and the group of newly colonising species in each province. To this end, we first created a Euclidean distance matrix based on normalised values of the four continuous numeric species traits (body size (wingspan), range size, mean and range of the species temperature index (STI); for details see ‘Species traits’ above). The trait distributions for the original communities and newly colonised species for a subset of the provinces (spanning the latitudinal range of the study area) were visualised with non-metric multidimensional scaling (nMDS) plots (created with the ‘metaMDS’ function in the ‘vegan’ package^119^).

The distance matrix was subsequently analysed with PERMANOVA (‘adonis’ function in the ‘vegan’ package^119^) to test for differences in average multidimensional trait distributions. To test for an overall effect, PERMANOVA was first run based on data for all provinces using 9999 permutations, grouping samples based on whether they represented the original community or the newly colonising species, and using province as a random factorial factor. To further evaluate in how many, and which, of the provinces that the trait distributions differed, PERMANOVA was also run separately for each of the 51 provinces (using 9999 permutations, and grouping samples based on whether they represented the original community or newly colonising species). *P*-values were FDR (false discovery rate) corrected with the method by Benjamini and Hochberg (1995)^120^ to account for multiple testing.

To test whether within-group variability of trait values differed between the species in the original community and newly colonising species, the PERMDISP implementation (‘betadisper function’) in the ‘vegan’ package was used to test for homogeneity of multivariate dispersions from centroids. As for PERMANOVA, PERMDISP was also run first on data for all provinces, and subsequently for each province separately, using sample grouping based on whether they represented the original community or the newly colonising species.

To investigate whether trait variability in the original community and newly colonising species were associated with latitude and province area, we ran two LMMs - one for species in the original community and one for the newly colonising species. For this, the average multivariate dispersion from centroid (proxy for within-group variability) was used as a continuous numeric response variable, and latitude (of province centroid) and province area as continuous numeric fixed predictor variables. To further evaluate whether the associations between within-group variability and latitude differed between the species in the original community and the newly colonising species, an additional LMM was run in which the two datasets were combined. As in the separate analyses, the average multivariate dispersion from centroid was used as a continuous numeric response variable; however, the model tested for an interaction effect between latitude (of province centroid, continuous numeric predictor variable) and species grouping (factorial predictor variable: original or colonised).

### Reporting summary

Further information on research design is available in the Nature Portfolio Reporting Summary linked to this article.

## Supplementary information

Supplementary Information

Description of Additional Supplementary Files

Supplementary Data 1

Supplementary Data 2

Reporting Summary

## Data Availability

All data used in this study was retrieved from open sources that are publicly available. Data on species distributions was retrieved from the national province level catalogue of Finnish and Swedish butterflies: 1901^90–96^, online sources (https://www.perhoset.fi and https://laji.fi/en), and Kullberg (2002)^97^; species traits from Eliasson, et al. (2005)^98^. Henriksen and Kreutzer (1982)^99^, Schweiger, et al. (2014)^100^, temperature data (HadCRUT) from Osborn, et al. (2021)^101^; and land cover data from the Historic Land Dynamics Assessment (HILDA) version 2.0 and the HILDA+ project^102–104^ (see Methods for details). A data file describing the compiled datasets used in this study is available as a supplementary data table (Supplementary Data 1), data used to create boxplots is available as a supplementary data file (Supplementary Data 2), and the compiled data files are available upon request.

## Code availability

R code is available upon request.
