# Supplementary material for: Century-long butterfly range expansions in northern Europe depend on climate, land use and species traits
Source: Commun Biol. 2023 Jun 3;6:601. doi: 10.1038/s42003-023-04967-z (PMC10239521; doi:10.1038/s42003-023-04967-z)
Supplement: Supplementary file 1 — Supplementary Information [file 42003_2023_4967_MOESM1_ESM.pdf]

Supplementary materials for

Century-long butterfly range expansions in northern Europe depend on climate, land use and species traits

Johanna Sunde[1], Markus Franzén[1], Per-Eric Betzholtz[1], Yannick Francioli[1], Lars B. Pettersson[2], Juha Pöyry[3], Nils Ryrholm[4] & Anders Forsman[1]*

[1] Department of Biology and Environmental Science, Linnaeus University, SE-39182 Kalmar, Sweden

[2] Biodiversity Unit, Department of Biology, Lund University, SE-22362 Lund, Sweden

[3] Finnish Environment Institute (SYKE), Nature Solutions, Latokartanonkaari 11, FI-00790 Helsinki, Finland,

[4] Department of Electronics, Mathematics and Natural Sciences, Faculty of Engineering and Sustainable Development, University of Gävle, SE-80176 Gävle, Sweden

[*]Corresponding author

Supplementary tables

Table S1. Associations of provincial colonisation rate of butterflies with time period, land use and temperature change. Results (output from ‘summary’ function) from a generalised linear mixed model (GLMM) for associations of variation in provincial colonisation rate (number of new species per province and decade) with time period, land use and temperature change. All predictor variables were normalised (with the ‘scale’ function in base R) to enable comparisons of slope estimates among the predictors.

Predictor	Estimate	Std. Error	z value	*P*-value	
(Intercept)	-34.03	12.86	-2.65	0.008	**
Forest	0.02	0.16	0.12	0.907	
Grassland	-0.02	0.17	-0.10	0.918	
Temperature change (linear)	-30.70	12.00	-2.56	0.011	*
Temperature change (squared)	-28.65	10.17	-2.82	0.005	**
Human settlements	0.19	0.22	0.86	0.392	
period1987	92.24	36.16	2.55	0.011	*
period2009	35.30	25.80	1.37	0.171	
period2019	34.43	12.86	2.68	0.007	**
Forest x Period1987	0.70	0.41	1.71	0.087	.
Forest x Period2009	0.59	0.33	1.78	0.075	.
Forest x Period2019	-0.32	0.23	-1.38	0.169	
Grassland x Period1987	0.73	0.44	1.65	0.099	.
Grassland x Period2009	0.29	0.34	0.84	0.399	
Grassland x Period2019	-0.31	0.27	-1.12	0.263	
Temperature change (linear) x Period1987	100.75	43.38	2.32	0.020	*
Temperature change (squared) x Period1987	47.70	15.91	3.00	0.003	**
Temperature change (linear) x Period2009	30.31	20.89	1.45	0.147	
Temperature change (squared) x Period2009	27.76	11.57	2.40	0.016	*
Temperature change (linear) x Period2019	29.69	12.01	2.47	0.013	*
Temperature change (squared) x Period2019	28.12	10.18	2.76	0.006	**
Human settlements x Period1987	-0.09	0.37	-0.23	0.815	
Human settlements x Period2009	-0.19	0.30	-0.64	0.523	
Human settlements x Period2019	-0.65	0.28	-2.34	0.020	*

Table S2. Associations of establishment success (range expansion) in butterflies with species traits. Results (output from ‘summary’ function) from a general linear mixed model for associations of variation in establishment success (number of new provinces colonised) with species traits. The four continuous numeric predictor variables (diet specialisation, body size (wingspan), and the mean and range of species temperature index) were normalised (with the ‘scale’ function in base R) to enable comparisons of slope estimates among the predictors.

Predictor	Estimate	Std. Error	z value	*P*-value	
(Intercept)	1.02	0.62	1.65	0.099	.
Diet specialisation (oligophage)	0.12	0.23	0.50	0.617	
Diet specialisation (polyphage)	-0.37	0.29	-1.27	0.203	
Body size (wingspan)	0.05	0.08	0.63	0.530	
Range size	0.46	0.12	3.88	< 0.001	***
Temperature range	0.73	0.20	3.62	< 0.001	***
Mean temperature	0.74	0.20	3.74	< 0.001	***
Habitat preference (generalist)	-0.56	0.32	-1.712	0.086	.
Habitat preference (openland)	-0.68	0.32	-2.10	0.036	*

Supplementary figures

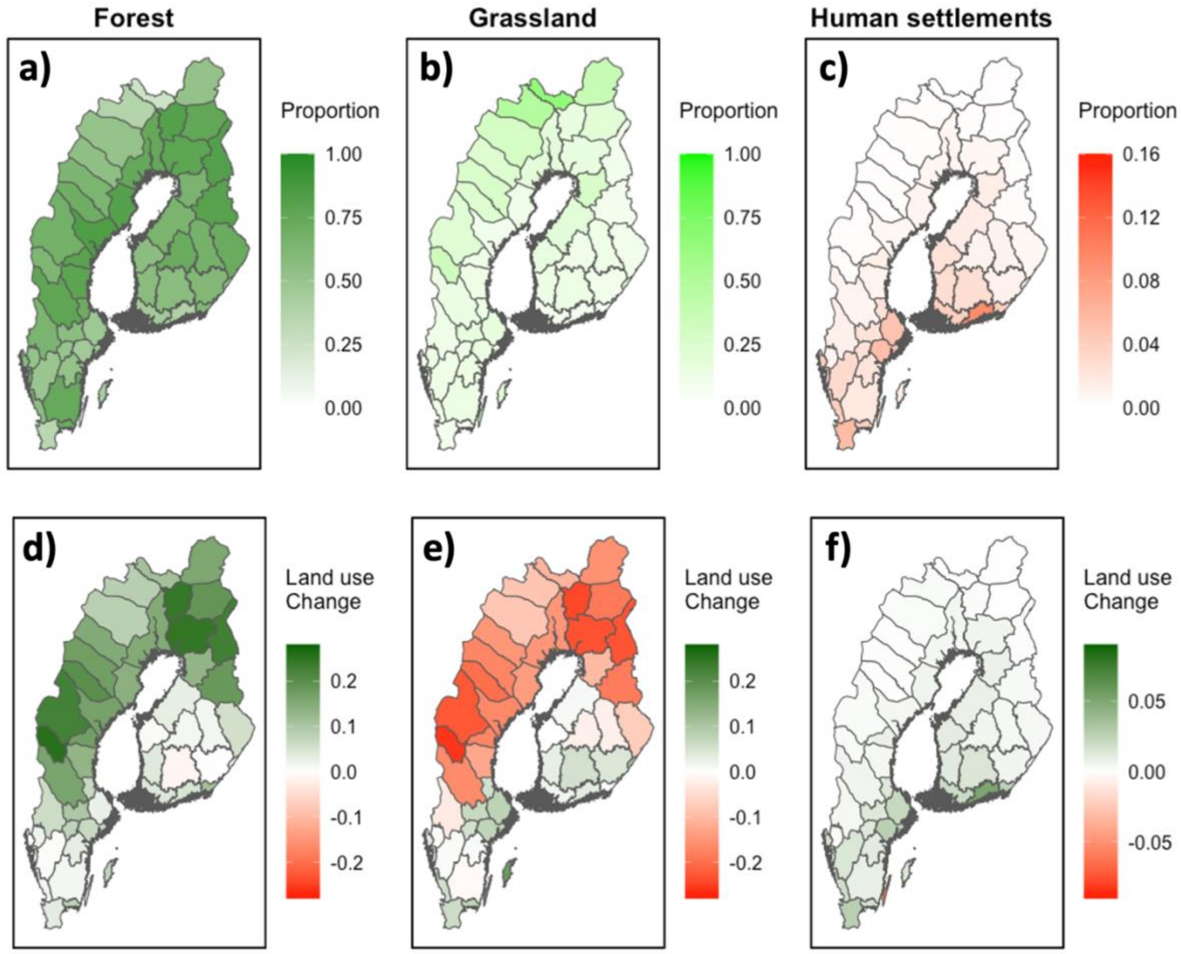

Figure S1. Land use and land use change in the 51 provinces in Sweden and Finland. The top row shows the proportion of the different land cover types (a) forest cover, b) grassland cover, and c) human settlements) in the 51 provinces in 2019. The bottom row shows land use change for each province from 1900 to 2019 for the three habitat types that have significantly changed ($P < 0.05$): d) change in forest cover, e) change in grassland cover, and f) change in human settlements.

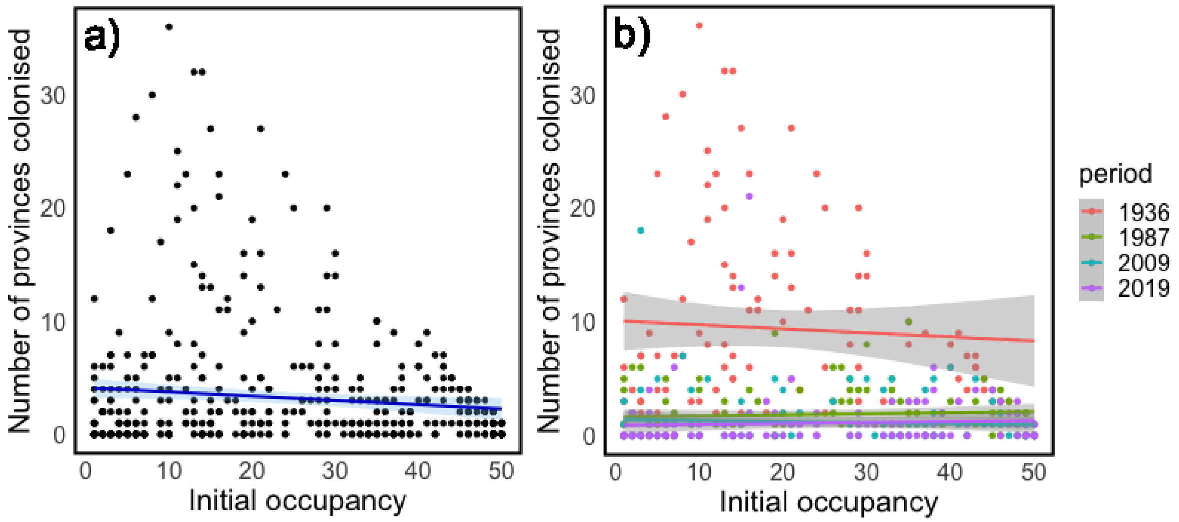

Figure S2. Figures show the relationship between the initial occupancy (number of provinces initially occupied by a species) and establishment success (number of new provinces colonised). a) shows the overall association when all values were pooled ($\chi 2$ = 0.57, *df* = 1, *n* = 467, *P* = 0.45), and b) shows how the number of colonised provinces is associated with the original occupancy for each species and time period (1901 - 1936, 1936 - 1987, 1987 - 2009, and 2009 - 2019) (interaction initial occupancy x period: $\chi 2$ = 2.41, *df* = 3, *n* = 467, *P* = 0.49). Each dot represents one species and one period, and trend lines represent regreson lines with 95% confidence intervals a) for the entire dataset, and b) estimated separately for each of the four time periods.

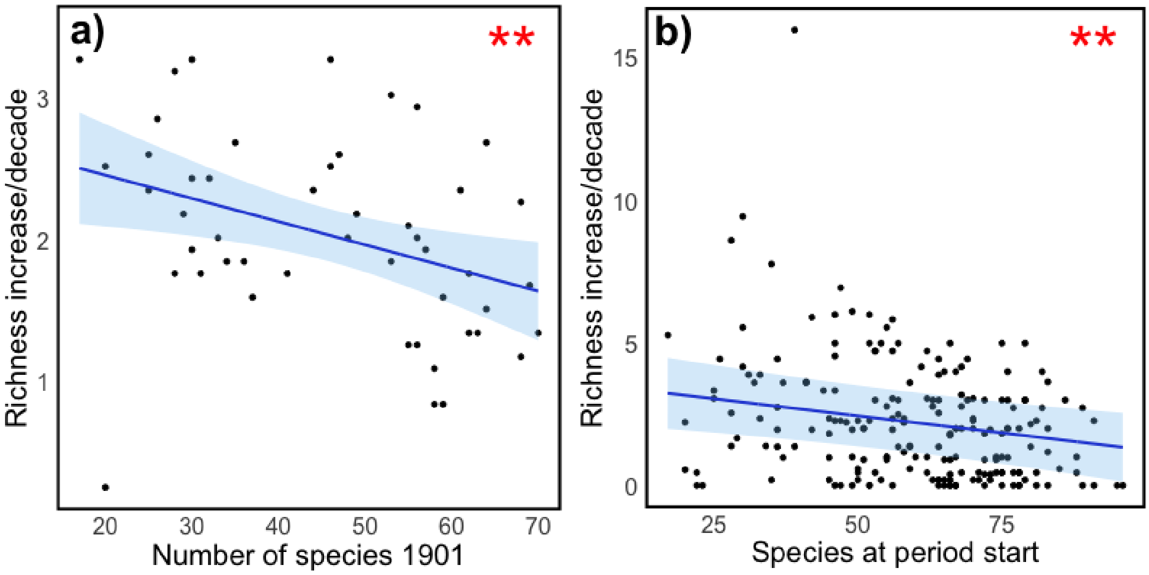

Figure S3. Association of increase in species richness with original species richness. a) shows the number of new species coloning each province per decade during the entire study time range (from 1901-2019) as a function of original species richness in each province (χ^2 = 7.44, *df* = 1, *n* = 48, *P* = 0.006). Each dot represents one province. b) shows how the number of coloning species per decade is associated with the original species richness in each province and time period (1901 - 1936, 1936 - 1987, 1987 - 2009, and 2009 - 2019) (χ^2 = 9.05, *df* = 1, *n* = 201, *P* = 0.003). Each dot represents one province and one time period. For both a) and b), the trend lines represent regression lines with 95% confidence intervals.

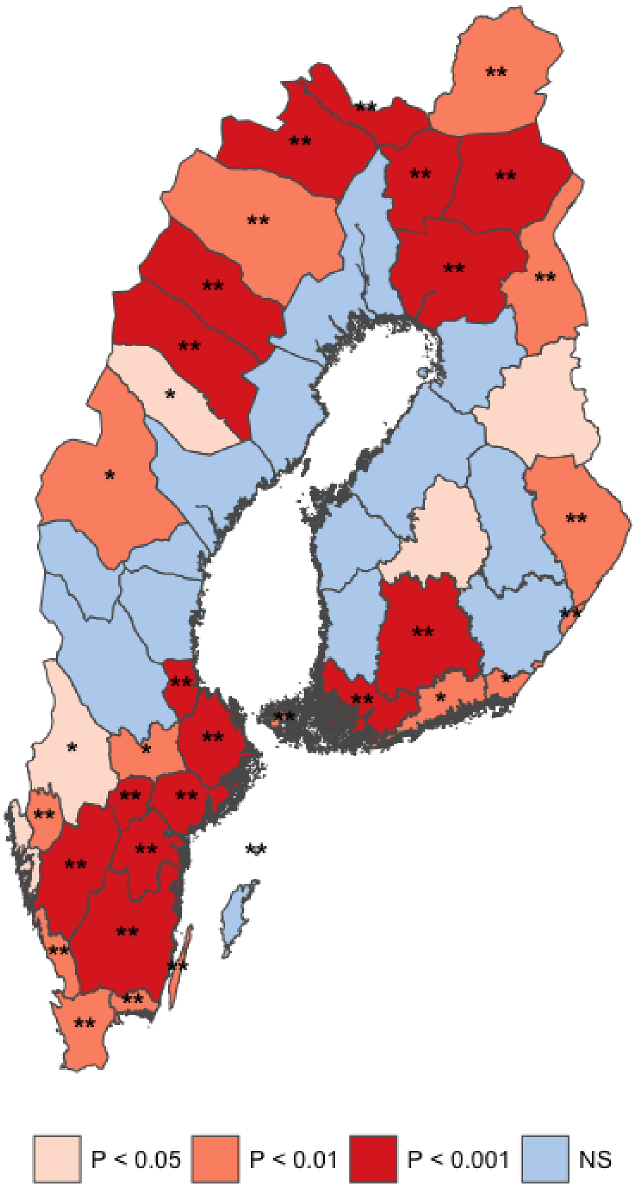

Figure S4. Pairwise comparisons of ecological trait values between species in the original community and colonising species. The figure shows test results for the 51 provinces. Colours in the map indicate results from separate PERMANOVA runs for each province testing for differences in species traits (the four continuous numeric variables: range size, body size (wingspan), and the mean and range of species temperature index) between original species in the provinces and species that have colonised the province after 1901. Shades of red indicate significant differences and blue non-significant results. Pairwise comparisons revealed that mean trait distributions were significantly different ($P < 0.05$) in 37 of the 51 (73%) provinces, and 34 of these remained significant after adjusting for multiple comparisons with fdr (q-value < 0.05). Adjusted *P*-values are indicated by stars.

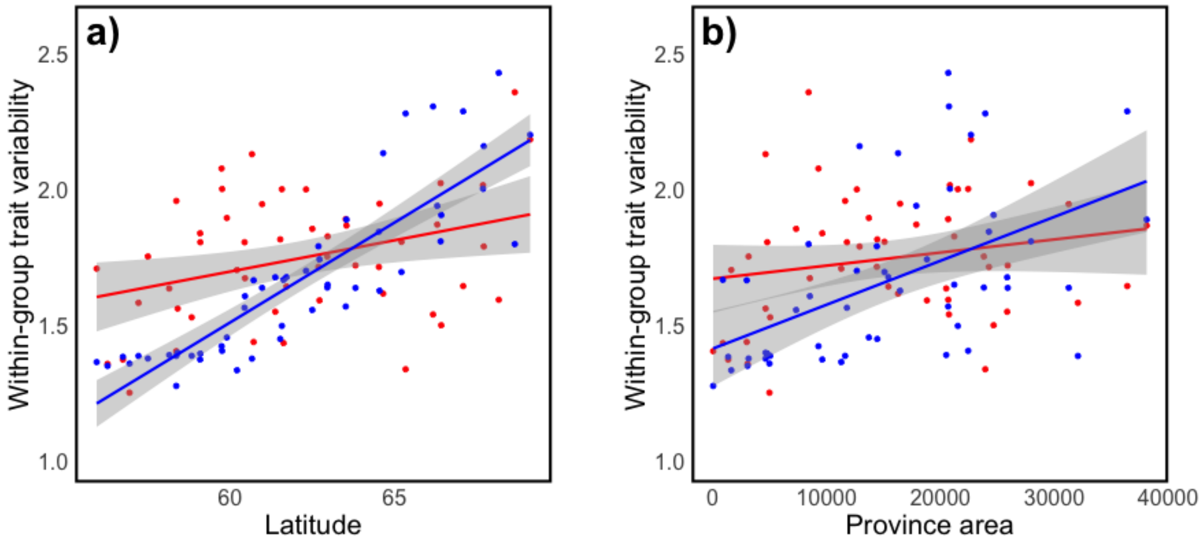

Figure S5. Within-group trait variability in original communities and in newly colonising species according to latitude and province size. a) shows the association between within-group trait variability and latitude, and b) the association between within-group trait variability and province area. Within-group variability was estimated as the dispersion from the centroid based on data of the continuous numeric variables body size (wingspan), species mean temperature, species temperature range, and distribution range size (using the PERMDISP implementation (disper function) in the vegan package in R). Each province contributed two values (dots), one for the original community (indicated in blue) and one for newly colonising species (indicated in red). Trend lines represent regression lines with 95% confidence intervals estimated separately for each of the two groups.

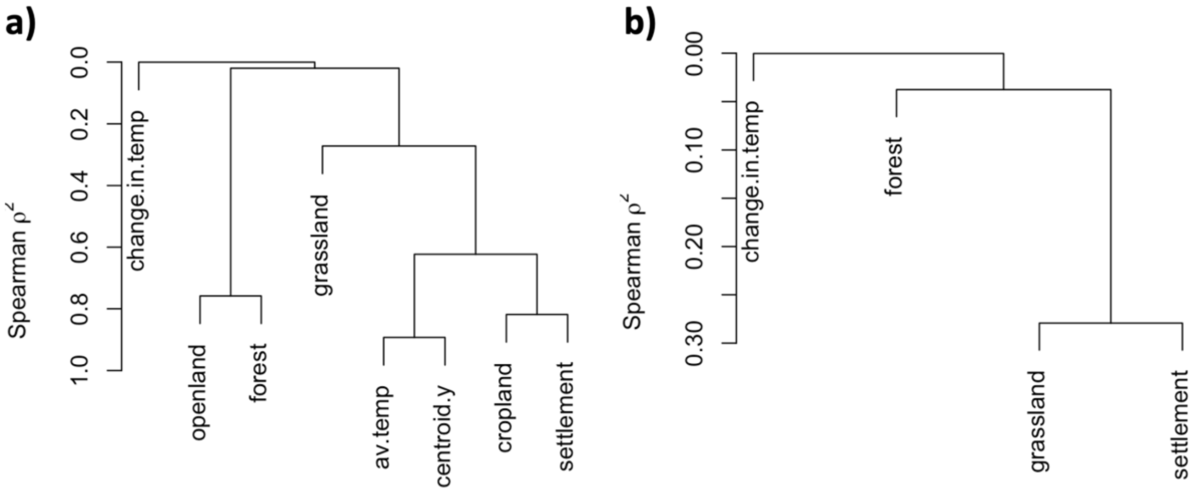

Figure S6. Cluster analysis based on Spearman correlation coefficient. Inclusion threshold was set to 0.3, which resulted in that of the eight initially included variables open land (openland), cropland cover (cropland), forest cover (forest), grassland cover (grassland), human settlements (settlement), average temperature (av.temp), temperature change (change.in.temp), and latitude (centroid.y); as seen in panel a), only four (forest cover, grassland cover, human settlements, and temperature change, as seen in panel b) were retained for analyses of land use.

Supplementary Note 1

To determine with certainty that a species has gone extinct from a province requires repeated visits to the province during the flight period of the species during suitable weather conditions. Also, species could go locally extinct and recolonise a province without this being noticed. Thus, it is virtually impossible to compile a scientifically reliable record over provincial extinctions. Anyway, we tried to compile a list over the species that possibly had gone extinct from one or some of the provinces. We found that 42 species represented by 184 province records could potentially be locally extinct from a province. This is only a very small fraction of all province records, 5% (184/3574). When analysing the data excluding the species categorised as locally extinct, the results remain qualitatively unchanged. When excluding the species categorised as locally extinct, the total species richness at start and end (108 – 131), the mean provincial species richness at both start and end (46 and 67, respectively), the provincial species richness increase (3 - 39, mean 21) and the percent species richness increase (15-218%, mean 58%) all remained the same or similar. The corresponding values when no potential local extinctions were excluded were: total species richness 108 - 131, mean species richness 46 and 70 (start and end), species richness increase 3 - 39 (mean 24), and percent species richness increase 15-229% (mean 64%).

In addition, regardless of whether data for species categorised as locally extinct was excluded or included in the analyses, species richness was negatively correlated with latitude (with potential extinctions excluded: $\chi2 = 13.65$, df = 1, $P < 0.001$) and positively correlated with temperature (with potential extinctions excluded: $\chi2 = 13.01$, df = 1, $P < 0.001$), and species richness increase was positively correlated with latitude (with potential extinctions excluded: $\chi2 = 18.25$, df = 1, $P < 0.0001$) and temperature (with potential extinctions excluded: $\chi2 = 13.98$, df = 1, $P < 0.001$).

Table S3. Summary table showing the number of provinces from where a species possibly has gone locally extinct for each of the five time points.

Species	Time	Num provinces
Aricia nicias	1987	1
Boloria chariclea	1987	2
Boloria freija	2019	2
Boloria frigga	1987	1
Boloria frigga	2009	2
Boloria napaea	2009	1
Boloria polaris	1987	1
Boloria polaris	2009	1
Boloria titania	1987	3
Boloria titania	2009	1
Carterocephalus palaemon	1936	1
Carterocephalus palaemon	1987	1
Carterocephalus palaemon	2019	1
Coenonympha glycerion	1987	1
Coenonympha hero	1987	3
Cupido minimus	1987	4
Cupido minimus	2009	2
Cyaniris semiargus	1936	1
Erebia embla	2009	3
Erebia pandrose	1900	1
Erebia pandrose	1936	1
Erebia pandrose	1987	1
Euphydryas aurinia	1987	5
Euphydryas maturna	1987	6
Fabriciana niobe	1987	8
Glaucopsyche alexis	1987	3
Glaucopsyche alexis	2009	2
Hamearis lucina	1987	4
Hesperia comma	1987	8
Hipparchia semele	1987	1
Hyponephele lycaon	1936	1
Issoria lathonia	1936	1
Issoria lathonia	1987	4
Lopinga achine	1987	3
Lopinga achine	2009	1
Lycaena helle	1987	16
Lycaena helle	2009	3
Lycaena hippothoe	1987	2
Lycaena hippothoe	2009	1
Lycaena phlaeas	1987	1
Lycaena tityrus	2019	1
Maniola jurtina	1987	4
Maniola jurtina	2009	1
Melitaea athalia	2009	1
Melitaea britomartis	1987	6
Melitaea cinxia	1987	3
Melitaea cinxia	2009	1
Melitaea diamina	1936	1
Melitaea diamina	1987	2
Melitaea diamina	2009	1
Oeneis norna	1900	1
Oeneis norna	2009	1
Parnassius apollo	1987	17
Parnassius apollo	2009	1
Parnassius mnemosyne	1987	8
Phengaris arion	1900	1
Phengaris arion	1936	1
Phengaris arion	1987	8
Phengaris arion	2009	2
Pseudophilotes vicrama	1987	5
Pyrgus alveus	1987	6
Pyrgus alveus	2009	1
Pyrgus centaureae	1987	2
Satyrium ilicis	1987	2
Satyrium pruni	1987	2
