# Supplementary material for: Century-long butterfly range expansions in northern Europe depend on climate, land use and species traits
Source: Commun Biol. 2023 Jun 3;6:601. doi: 10.1038/s42003-023-04967-z (PMC10239521; doi:10.1038/s42003-023-04967-z)
Supplement: Supplementary file 2 — Description of Additional Supplementary Files [file 42003_2023_4967_MOESM2_ESM.pdf]

Description of Additional Supplementary Files

File Name: Supplementary Data 1
Description: Description of public datasets used in this study.

File Name: Supplementary Data 2
Description: Source data used to create boxplots in Figures 3-5.
