# Supplementary material for: Century-long butterfly range expansions in northern Europe depend on climate, land use and species traits
Source: Commun Biol. 2023 Jun 3;6:601. doi: 10.1038/s42003-023-04967-z (PMC10239521; doi:10.1038/s42003-023-04967-z)
Supplement: Supplementary file 3 — Supplementary Data 1 [file 42003_2023_4967_MOESM3_ESM.pdf]

Dataframe	Description of dataframe	Variable	Description of variable
**df.clim.hex.mean.Rdata**	hexagon temperature data	country	country
		x	longitudinal position of hexagon centroid
		y	latitudinal position of hexagon centroid
		xmin	minimum longitude of hexagon
		ymin	minimum latitude of hexagon
		xmax	maximum longitude of hexagon
		ymax	maximum latitude of hexagon
		temp.1901	start temperature (mean annual temperature during 1900-1909)
		temp.2019	end temperature (mean annual temperature 2010-2019)
**df.overall.climate.tracking.Rdata**	climate tracking data (overall 1901-2019)	species.number	species identifier
		tot.dist.north.per.year	rate of northward species shift over the entire study timespan (1901-2019) (northward shift in km per year)
		tot.dist.clim.north.per.year	rate of northward temperature shift over the entire study timespan (1901-2019) (northward shift in km per year)
**df.period.climate.tracking.Rdata**	climate tracking data (period-based)	species.number	species identifier
		period	study period (1936 = 1901-1936; 1987 = 1936-1987; 2009 = 1987-2009; 2019 = 2009-2019)
		dist.north.per.year	rate of northward species shift (northward shift in km per year)
		dist.clim.north.per.year	rate of northward temperature shift (northward shift in km per year)
**df.landuse.decade.prov.long.Rdata**	land use data (decades)	province	province abbreviation
		year	year
		habitat	habitat/land type category (one on forest, grassland, or settlement)
		prop	proportional coverage of the habitat/land type category
**df.landuse.timepoints.prov.long.Rdata**	land use data (timepoints)	province	province abbreviation
		timepoint	year of the timepoint (one of: 1901, 1936, 1987, 2009, 2019)
		habitat	habitat/land type category (one on forest, grassland, or settlement)
		prop	proportional coverage of the habitat/land type category
**df.traits.colonised.period.Rdata**	species trait data	species.number	species identifier
		family.number	family identifier
		period	study period (1936 = 1901-1936; 1987 = 1936-1987; 2009 = 1987-2009; 2019 = 2009-2019)
		prov.colonized	species establishment success (number of provinces colonized during the period)
		num.provinces.initial	species initial occupancy (number of provinces inhabited at the start of the period)
		habitat.preference	species habitat preference (one of: open, forest, or generalist)
		diet.specialisation	larval dietary breadth of the species (1 = monophagous, 2= oligophagous, and 3 = polyphagous)
		body.size.wing	adult body size of the species (male wingspan in mm)
		range.size	species range size (number of European 50 x 50 km grid cells occupied)
		ann.temp.mean	mean of species temperature index (in °C)
		range.ann.temp.mean	range of species temperature index (difference, in °C, between the warmest the coldest month)
**df.traits.new.old.norm.Rdata**	species trait data for original community and newly colonising species	species.number	species identifier
		province	province abbreviation
		centroid.y	latitudinal position of province centroid
		new.or.old	information about whether the species was part of the original community ('original') or newly colonizing species ('colonised')
		body.size.wing.norm	normalised value of the species' adult body size
		range.size.norm	normalised value of the species' range size
		ann.temp.mean.norm	normalised value of the species' mean species temperature index
		range.ann.temp.mean.norm	normalised value of the species' range of species temperature index

Dataframe	Description of dataframe	Variable	Description of variable
**shp.prov.overall.Rdata**	shapefile with overall (1901-2019) data	province	province abbreviation
		full.name	province name
		centroid.x	longitudinal position of province centroid
		centroid.y	latitudinal position of province centroid
		ext.min.x	minimum longitude of province
		ext.max.x	maximum longitude of province
		ext.min.y	minimum latitude of province
		ext.max.y	maximum latitude of province
		start.temp	start temperature (mean annual temperature during 1900-1909)
		end.temp	end temperature (mean annual temperature 2010-2019)
		temp.increase	overall temperature increase, in °C, during the entire study timespan (1901-2019)
		start.species	original species richness (number of species in the province in 1901)
		nb.species	cumulative species richness (cumulative number of butterfly species observed in the province)
		species.increase	overall species richness increase during the entire study timespan (1901-2019) (number of new species in the province)
		species.increase.per.year	rate of species richness increase (number of new species per year during the entire study timespan (1901-2019))
		vector.species.x	end longitude for provincial species shift
		vector.species.y	end latitude for provincial species shift
		angle.species	angle of provincial species shift
		angle.clim	angle of provincial temperature shift
		pos	spatial structure variable (long, lat)
		group	dummy variable for random structure in spatial structure construction
		vector.x	end longitude for provincial temperature shift
		vector.y	end latitude for provincial temperature shift
		centroid.x.clim	start longitude for provincial temperature shift (longitudinal position of centroid for province temperature based on hexagon data)
		centroid.y.clim	start latitude for provincial temperature shift (latitudinal position of centroid for province temperature based on hexagon data)
		area	province area
		geometry	geometry
**shp.prov.period.Rdata**	shapefile with period-based data	province	province abbreviation
		period	study period (1936 = 1901-1936; 1987 = 1936-1987; 2009 = 1987-2009; 2019 = 2009-2019)
		av.temp	temperature (average temperature during the last 10 years of the period)
		centroid.x.clim	start longitude for provincial temperature shift (longitudinal position of centroid for province temperature based on hexagon data)
		centroid.y.clim	start latitude for provincial temperature shift (latitudinal position of centroid for province temperature based on hexagon data)
		vector.x	end longitude for provincial temperature shift
		vector.y	end latitude for provincial temperature shift
		nb.species	cumulative species richness (cumulative number of butterfly species observed in the province up to the timepoint)
		centroid.x	longitudinal position of province centroid
		centroid.y	latitudinal position of province centroid
		vector.species.x	end longitude for provincial species shift
		vector.species.y	end latitude for provincial species shift
		angle.species	angle of provincial species shift
		angle.clim	angle of provincial temperature shift
		start.species	original species richness (number of species in the province at the beginning of the period)
		colonised.per.decade	provincial colonisation rate (new species per decade)
		geometry	geometry

Dataframe	Description of dataframe	Variable	Description of variable
**shp.prov.overall.landuse.Rdata**	shapefile with overall (1901-2019) land use data	province	province abbreviation
		full.name	province name
		centroid.x	longitudinal position of province centroid
		centroid.y	latitudinal position of province centroid
		ext.min.x	minimum longitude of province
		ext.min.y	minimum latitude of province
		ext.max.x	maximum longitude of province
		ext.max.y	maximum latitude of province
		settlement	propotion of human settlements
		grassland	propotion of grassland cover
		forest	propotion of forest cover
		forest.diff	change in forest cover (difference in proportional cover from 1901 to 2019)
		grassland.diff	change in grassland cover (difference in proportional cover from 1901 to 2019)
		settlement.diff	change in human settlements (difference in proportional cover from 1901 to 2019)
		geometry	geometry
**shp.prov.period.landuse.Rdata**	shapefile with period-based landuse data	province	province abbreviation
		period	study period (1936 = 1901-1936; 1987 = 1936-1987; 2009 = 1987-2009; 2019 = 2009-2019)
		colonised.per.decade	provincial colonisation rate (new species per decade)
		forest	propotion of forest cover
		grassland	propotion of grassland cover
		temp.change.per.decade	rate of temperature change (°C per decade)
		settlement	propotion of human settlements
		openland	propotion of open land
		cropland	propotion of cropland
		av.temp	temperature (average temperature during the last 10 years of the period)
		change.in.temp	change in temperature (difference, in °C, in temperature during the period)
		centroid.y	latitudinal position of province centroid
		geometry	geometry
