# Supplementary material for: Century-long butterfly range expansions in northern Europe depend on climate, land use and species traits
Source: Commun Biol. 2023 Jun 3;6:601. doi: 10.1038/s42003-023-04967-z (PMC10239521; doi:10.1038/s42003-023-04967-z)
Supplement: Supplementary file 5 — Reporting Summary [file 42003_2023_4967_MOESM5_ESM.pdf]

nature portfolio

Corresponding author(s): Johanna Sunde

Last updated by author(s): May 23, 2023

Reporting Summary

Nature Portfolio wishes to improve the reproducibility of the work that we publish. This form provides structure for consistency and transparency in reporting. For further information on Nature Portfolio policies, see our Editorial Policies and the Editorial Policy Checklist.

Statistics

For all statistical analyses, confirm that the following items are present in the figure legend, table legend, main text, or Methods section.

n/a	Confirmed	
☐	☒	The exact sample size (n) for each experimental group/condition, given as a discrete number and unit of measurement
☒	☐	A statement on whether measurements were taken from distinct samples or whether the same sample was measured repeatedly
☐	☒	The statistical test(s) used AND whether they are one- or two-sided<br>*Only common tests should be described solely by name; describe more complex techniques in the Methods section.*
☐	☒	A description of all covariates tested
☐	☒	A description of any assumptions or corrections, such as tests of normality and adjustment for multiple comparisons
☐	☒	A full description of the statistical parameters including central tendency (e.g. means) or other basic estimates (e.g. regression coefficient) AND variation (e.g. standard deviation) or associated estimates of uncertainty (e.g. confidence intervals)
☐	☒	For null hypothesis testing, the test statistic (e.g. F, t, r) with confidence intervals, effect sizes, degrees of freedom and P value noted<br>*Give P values as exact values whenever suitable.*
☒	☐	For Bayesian analysis, information on the choice of priors and Markov chain Monte Carlo settings
☒	☐	For hierarchical and complex designs, identification of the appropriate level for tests and full reporting of outcomes
☒	☐	Estimates of effect sizes (e.g. Cohen's d, Pearson's r), indicating how they were calculated

Our web collection on statistics for biologists contains articles on many of the points above.

Software and code

Policy information about availability of computer code

Data collection

All data used in this study was collated from open sources: temperature from Osborn et al. (2021), land cover from Fuchs et al. (2013), Fuchs et al. (2015a), Fuchs et al. (2015b), species distributions from Aro (1900), Aurivillius (1891), Grönblom (1936), Gustafsson (2019), Kullberg (2002), Lampa (1885), Nordström (1943), Sotavalta (1987), and species traits (diet breadth from, body size, range size, mean and range of species temperature index (STI), and habitat preference) from Eliasson et al. (2005), Henriksen & Kreutzer (1982), Schweiger et al. (2014).

References

Aro, JE, Suomen Perhoset. 1900, Helsinki, Finland: Otava.

Aurivillius, C, Nordens Fjärilar. 1891, Stockholm, Sweden: Hierta.

Eliasson, CU, et al., Nationalnyckeln till Sveriges flora och fauna. Fjärilar: Dagfjärilar. Hesperidae - Nymphalidae. 2005, Uppsala: ArtDatabanken, SLU.

Fuchs, R, et al., Biogeosciences, 2013. 10(3): p. 1543-1559.

Fuchs, R, et al., Glob. Change Biol., 2015a. 21(1): p. 299-313.

Fuchs, R, et al., Applied Geography, 2015b. 59: p. 43-55.

Grönblom, T, Acta societatis pro fauna et flora Fennici, 1936. 58: p. 1-45.

Gustafsson, B, Catalogus Lepidopterorum Sueciae. 2019, Swedish Museum of Natural History: Stockholm.

Henriksen, HJ and IB Kreutzer, The butterflies of Scandinavia in nature. 1982, Odense: Skandinavisk Bogforlag. 1-215.

Kullberg, J, Sahlbergia, 2002. 6: p. 45-190.

Lampa, S, Entomologisk Tidskrift, 1885. 6: p. 1-137.

Nordström, F, Opusc. Ent., 1943. 8: p. 59-120.

Osborn, TJ, et al., Journal of Geophysical Research: Atmospheres, 2021. 126(2): p. e2019JD032352.

Schweiger, O, et al., ZooKeys, 2014. 367.

Sotavalta, O, Notulae Entomologicae, 1987. 67: p. 187-205.

Data analysis	In the present study, all statistical analyses were carried out in RStudio2 v.1.3.1093 RStudio Team, in R.4.0.3 R Core Team, 2021, using the following packages: 'ggplot' Wickham, 2016 for data visualisations, 'glmmTMB' Brooks et al., 2017 for GLMMs, 'car' Fox & Weisberg, 2019 for type III Likelihood Ratio tests, 'drc' Ritz et al., 2015 for asymptotic regression analysis, 'ggpubr' Kassambra, for calculating Pearson correlation coefficients, 'circular' Agostinelli & Lund, 2017 for Watson-Williams tests, 'Hmisc' Harrell, 2020 for Varclus analysis, 'effects' Fox & Weisberg, 2019 for obtaining predicted means, 'emmeans' to calculate least-squares means, 'vegan' Oksanen et al., 2019 for nMDS plotting, PERMANOVA and PERMDISP, and tidyverse Wickham et al., 2019 for data wrangling. R code is available upon request.<br><br>Agostinelli, C and U Lund, R package "circular": circular statistics. 2017.<br>Brooks, ME, et al., The R journal, 2017. 9(2): p. 378-400.<br>Fox, J and S Weisberg, An R companion to applied regression. 2019, Thousand Oaks, CA: Sage publications.<br>Harrell, FE, Hmisc: Harrell Miscellaneous. 2020.<br>Kassambra, A, ggpubr: 'ggplot2' Based Publication Ready Plots. 2020.<br>Lenth, RV, emmeans: Estimated Marginal Means, aka Least-Squares Means. 2022.<br>Oksanen, J, et al., vegan: Community Ecology Package. 2019.<br>R Core Team, R: A language and environment for statistical computing., in R Foundation for Statistical Computing. 2021: Vienna, Austria.<br>Ritz, C, et al., PLoS ONE, 2015. 10(12): p. e0146021.<br>RStudio Team, RStudio: integrated development environment for R. Boston, MA: RStudio, PBC; 2020.<br>Wickham, H, ggplot2: Elegant graphics for data analysis. 2016, Springer-Verlag: New York.<br>Wickham, H, et al., Journal of Open Source Software, 2019. 4(43).

For manuscripts utilizing custom algorithms or software that are central to the research but not yet described in published literature, software must be made available to editors and reviewers. We strongly encourage code deposition in a community repository (e.g. GitHub). See the Nature Portfolio guidelines for submitting code & software for further information.

Data

Policy information about availability of data

All manuscripts must include a data availability statement. This statement should provide the following information, where applicable:

- Accession codes, unique identifiers, or web links for publicly available datasets
- A description of any restrictions on data availability
- For clinical datasets or third party data, please ensure that the statement adheres to our policy

All data used in this study was retrieved from publicly available open sources (as detailed in the section 'data collection' above). Because of restrictions from the sources from which the raw data was retrieved, the compiled datasets will not be deposited in any public depository. The sources of the raw data is however clearly stated, and the compiled datasets are available upon request. In addition, a data file containing explanations of the compiled datasets used in this study is provided as a supplementary data table (Supplementary Data 1) to facilitate such requests, and the data used to create boxplots is available as a supplementary data file (Supplementary Data 2).

Field-specific reporting

Please select the one below that is the best fit for your research. If you are not sure, read the appropriate sections before making your selection.

☐ Life sciences ☐ Behavioural & social sciences ☒ Ecological, evolutionary & environmental sciences

For a reference copy of the document with all sections, see nature.com/documents/nr-reporting-summary-flat.pdf

Ecological, evolutionary & environmental sciences study design

All studies must disclose on these points even when the disclosure is negative.

Study description	We study the distribution and distributional changes of butterflies in Sweden and Finland during the last century in relation to temperature, land use, and species traits, to determine if butterflies have been able to track climate change and to evaluate whether and how the climate, environment and/or species traits influence climate tracking ability.
Research sample	Data on species presence/absence, temperature, and land use for 51 provinces, covering the entire Sweden and Finland, was collected for five timepoints (1901, 1936, 1987, 2009, and 2019). Species traits data includes information on a total of 130 butterfly species.
Sampling strategy	We sampled the provincial distribution of 131 butterfly species known to occur in Sweden and Finland in five time points 1901, 1936, 1987, 2009, and 2019) by data extraction from other sources (as specified in the "data collection" section above). We chose to include information on distributions for as many butterfly species as possible (all species occurring in the study area during the included timespan, Ntot = 131) . The choice of time points was based on data availability, and spans over a century. Information of species traits were collected for all species for which range shifts were possible (all but Aglais urticae (n = 130), which occurred in all provinces already at the first time point (1901).
Data collection	All data used in this study was collected from open sources (for details see the 'Data collection' section above).
Timing and spatial scale	We use climate data, land-cover data and distribution data of 131 species of butterflies at five timepoints (1901, 1936, 1987, 2009, and 2019) in 51 provinces covering the entire Sweden and Finland.

Data exclusions	To minimize the risk of data bias, taxa that had been split during the study period (n = 2, Plebejus argus and Plebejus idas, Leptidea sinapis and Leptidea juvernica) was merged to one taxa respectively (resulting in a total of 131 included species).<br><br>Data on species richness at the first time point and species richness increase during the first period were excluded for three of the Finish provinces (Om, Oa and St) due to inadequate sampling and province delimitations in the early 1900s.<br><br>In addition, one species (Aglais urticae) were excluded from the analysis evaluating whether species traits influenced colonization success because it occured in all provinces already at the first timepoint (i.e. could not colonize any new provinces).
Reproducibility	We describe the study to make it easy to follow and to reproduce. Raw data is publicly available in open sources. Compiled data files and R code used in the study is available upon request.
Randomization	Not applicable.
Blinding	Not applicable - blinding was not possible because of the type of the data used.

Did the study involve field work? ☐ Yes ☒ No

Reporting for specific materials, systems and methods

We require information from authors about some types of materials, experimental systems and methods used in many studies. Here, indicate whether each material, system or method listed is relevant to your study. If you are not sure if a list item applies to your research, read the appropriate section before selecting a response.

Materials & experimental systems

n/a	Involved in the study	
☒	☐	Antibodies
☒	☐	Eukaryotic cell lines
☒	☐	Palaeontology and archaeology
☒	☐	Animals and other organisms
☒	☐	Human research participants
☒	☐	Clinical data
☒	☐	Dual use research of concern

Methods

n/a	Involved in the study	
☒	☐	ChIP-seq
☒	☐	Flow cytometry
☒	☐	MRI-based neuroimaging
